# Online identity work dynamics of Instagram micro-influencers: an extreme case approach

**DOI:** 10.3389/fpsyg.2023.1306248

**Published:** 2023-12-14

**Authors:** Yoy Bergs, Pascale Peters, X. D. Lub, R. J. Blomme

**Affiliations:** ^1^Strategy, Organization and Leadership, Nyenrode Business Universiteit, Breukelen, Netherlands; ^2^Academy of Hotel and Facility Management, Breda University of Applied Sciences, Breda, Netherlands; ^3^Organisations in Digital Transformation, Utrecht University of Applied Sciences, Utrecht, Netherlands; ^4^Faculty of Management, Open Universiteit, Heerlen, Netherlands

**Keywords:** identity, virtual identity, online identity, dynamics, social media, extreme case analysis

## Abstract

**Background:**

Advanced media technologies have become an integral part of people's daily lives, providing them with new tools and environments for the formation and enactment of their identities. To date, the literature acknowledges that media technologies, such as social networking sites, are used to form and enact online identities, and that these platforms can simultaneously pose challenges to individuals' identity work. However, we know little about the precise online identity work strategies that individuals employ in response to the challenges they face over time.

**Objective:**

This paper examines the online identity work dynamics of Instagram micro-influencers, for whom social network sites enable and guide them in forming and enacting their online identities on a daily basis. The study was guided by the following research question: what are the challenges that Instagram micro-influencers perceive online and what are the online identity work strategies that they employ in response to these challenges over time?

**Methods:**

This study employs an extreme case approach to rigorously explore the lives of seven micro-influencers on Instagram. We combine in-depth data from narrative interviews, longitudinal data from online autobiographical narratives revealed through the participants' Instagram timelines, and follow-up interviews.

**Results:**

Our analysis revealed three main themes that highlight the challenges that Instagram micro-influencers face online: (1) amplified social expectations, (2) feelings of inauthenticity, and, as a result thereof, (3) psychological distress. We found that these challenges were viewed as catalysts for their online identity work processes. We identified three key online identity work strategies that the Instagram micro-influencers employed in response over time: (1) experimenting with their online identities, followed by either (2) segmenting between their online and offline identities, or (3) adding identities through online multiplicity.

**Conclusion:**

Our research provides new insights into how individuals may respond to the challenge of managing their online identities over time by engaging in different online identity work strategies. This study highlights the importance of designing online media technologies that enable individuals to cope with online challenges. We emphasize the need to design online spaces for (1) the expression of authentic identities, (2) community building, and (3) online multiplicity.

## 1 Introduction

Advanced media technologies are becoming increasingly ubiquitous and integral to people's daily lives, providing new tools and environments for the formation and enactment of their identities (Cover, 2015; Barros et al., 2023). In the current study, we define identity as the “individuals' subjective interpretations of who they are, based on their socio-demographic characteristics, roles, personal attributes, and group memberships” (Caza et al., 2018, p. 889). For example, an individual could define themselves as a female, daughter, introvert, marketeer, gymnast, and mountaineer. Each of the identities an individual holds exists within a network of people who may have particular expectations of the individual (Davis and Jurgenson, 2014). These expectations, in turn, inform the individual's identity work (Brown, 2021), defined as “the cognitive, discursive, physical, and behavioral activities that individuals undertake with the goal of forming, repairing, maintaining, strengthening, revising, or rejecting collective, role, and personal self-meanings” (Caza et al., 2018, p. 895). Consequently, identity work can be considered as a dynamic process in which identities are constantly being confirmed and modified in negotiation with others (Maitlis, 2009; Ramarajan, 2014; Caza et al., 2018; Bataille and Vough, 2022).

Online identity work is then seen as the process of identity work that takes place in virtual worlds, such as on social network sites (SNSs) (Boyd and Ellison, 2007; Cover, 2015; Barros, 2018; Barros et al., 2023). SNSs refer to the set of interactive internet applications that facilitate the creation and sharing of user-generated content (Davis, 2012). The most widely used platforms are Facebook, YouTube, and Instagram, all of which have more than one billion monthly active users worldwide (Statista, 2023). On these platforms, individuals form and enact their online identities in negotiation with others in their social networks (Marwick, 2013; Hafer et al., 2023).

Studies in the fields of communication (e.g., Davis and Jurgenson, 2014) and media technology (Carter and Grover, 2015; Shah and Tewari, 2016; Bishop, 2018) have focused on how media technologies are increasingly used for strategic self-presentational activities, image management, and self-branding (Senft, 2013). Specifically, apps such as Instagram allow individuals to upload pictures and videos of their lives to the platform and share them publicly (Abidin, 2016). In doing so, individuals can explore and create online narratives that further define who they are (Cover, 2015; Gu et al., 2022; Hafer et al., 2023). Given the ability on these platforms to conceal what one does not want to convey, or to accentuate what is important, SNSs allow individuals to present themselves in a socially desirable way (Ellison et al., 2006; Hafer et al., 2023). Specifically, via SNSs, individuals can form and enact identities online that reflect ideal personas. For example, through the presentation of filtered selfies (Halpern et al., 2017), the use of hashtags (Nasrin and Fisher, 2022), or profile pictures (Blanco Ramírez and Palu-ay, 2015).

While scholars in the fields of identity and communication and media technology have acknowledged that media technologies can provide individuals with new environments and tools for (1) exploring their identities (Stanko et al., 2019; Soini and Eräranta, 2023) and (2) publicly and strategically expressing these identities (Boyd, 2010; Marwick and Boyd, 2011; Bonneau et al., 2023), there is also emerging evidence that further highlights the severe challenges of engaging in online identity work.

First, individuals may experience identity conflicts as social demands from others increase due to the blending of their physical and virtual social networks (Davis and Jurgenson, 2014; Hafer et al., 2023). Specifically, when an individual experiences an inability to maintain and balance the expectations coming from different social contexts, this can trigger tensions between their different identities (i.e., identity conflicts) (Petriglieri, 2011; Ramarajan et al., 2017; Gibson et al., 2021).

Second, it has been argued that there is a greater need for individuals to present their authentic selves on online platforms (Marwick, 2013; Haimson and Hoffmann, 2016; Haimson et al., 2021). Authenticity refers to “the extent to which an individual acts in accord with the true self and it involves owning one's personal experiences, thoughts, emotions, needs, wants, preferences, or beliefs” (Roberts, 2005, p. 699). This means that individuals are required to present a consistent, positive, and “true” self across both their online and offline environments (Haimson et al., 2021). Online authenticity can be understood in two dimensions – a sense of a true self and the expression of a true self online (Lim et al., 2015). The first dimension refers to the degree to which one owns one's personal experiences, i.e., thoughts, values, emotions, and beliefs. The second dimension concerns whether one acts in accordance with one's true self online, that is, whether one expresses online what one thinks and believes (Gardner et al., 2005). However, even though individuals may increasingly strive to present themselves authentically on social media, research suggests that they often feel unable to do so (Haimson et al., 2021), also referred to as the authenticity paradox. Being authentic requires sharing negative experiences online and is therefore only possible at great personal cost (Ibarra, 2015; Haimson et al., 2021). In turn, the feeling that individuals are unable to act in accordance with their true selves on online platforms can lead to severe identity conflicts, which can potentially lead to detrimental mental health issues (Berryman et al., 2018), such as depression (Keles et al., 2020), anxiety (Primack et al., 2017), and burnout (Harren et al., 2021).

While the literature acknowledges that SNSs are used to form and enact identities (Davis, 2012; Cover, 2015; Haimson and Hoffmann, 2016), and can simultaneously pose challenges to online identity work (Davis, 2012; Halpern et al., 2017; Haimson et al., 2021), thus far, we know little about the exact online identity work strategies that individuals employ in response to these challenges. Moreover, identity work studies often take a rather static picture and tend to overlook dynamic elements (i.e., temporal patterns during alternating phases of identity work) (Vantilborgh et al., 2018). This is important as it may advance our understanding of *what* happens, *when* and *how* things happen. This paper explores this further by examining the identity work dynamics of those for whom SNSs enable and guide how they form and enact their online identities on a daily basis: Instagram micro-influencers (Abidin, 2016; Roccapriore and Pollock, 2022). Accordingly, the study was guided by the following research question: what are the challenges that Instagram micro-influencers perceive online and what are the online identity work strategies they employ in response to these challenges over time?

Overall, our study extends the literature on identity work and online identities in three ways. First, we show that online identity work is a dynamic process that changes over time. In particular, our longitudinal empirical study suggests that individuals initially enter a phase of experimentation in response to the challenges they face online. Here, social media platforms provide a safe liminal space for individuals to freely experiment with desired identities by employing online identity work tactics such as online activation cues and/or online relationship building (Stanko et al., 2019, 2022). Second, our paper contributes by highlighting two different pathways that individuals may choose over time to further cope with the challenges they experience. After a period of experimentation, individuals may either further segment their online and offline identities (Desrochers and Sargent, 2004; Ollier-Malaterre et al., 2013; Dumas and Sanchez-Burks, 2015), or add more identities through online multiplicity (Carter, 2008). Our findings on online multiplicity provide new insights into identity continuity (Wittman, 2019) and may provide answers to the experience of the online authenticity paradox (Haimson et al., 2021). As mentioned earlier, the online authenticity paradox is the experienced difficulty of presenting a consistent and true self across online and offline environments. We find that some individuals, despite feeling inauthentic online, may struggle to change their online identities because these online identities have become entrenched through both sustained social interactions with their followers and the need for online success (Banet-Weiser, 2012; Duffy and Hund, 2015; Abidin, 2016). In turn, adding identities through online multiplicity (Carter, 2008) may provide individuals with an opportunity to present a more consistent, positive, and “true” self without having to discard a certain status that was built within their initial account. Third, and finally, we make a methodological contribution by adopting an extreme case approach (Eisenhardt, 1989), combining narrative interviews with the analysis of online autobiographical narratives revealed through Instagram timelines, and follow-up interviews. The complementary data allowed us to explore the dynamic elements of online identity work by following Instagram micro-influencers over time.

## 2 Materials and methods

### 2.1 Research context

Micro-influencers are individuals with a social media presence larger than a “normal” person but smaller than a celebrity, ranging from 1,000 to 100,000 followers (Urwin, 2022). According to research from the Influencer Marketing Hub (2021), there are ~1.21 billion active Instagram influencers, the majority of which are micro-influencers with between 1,000 and 100,000 followers. However, there are also mega-influencers with millions of followers who can earn up to hundreds of thousands of dollars per post. The audience of micro-influencers is smaller but often highly engaged (Urwin, 2022).

Instagram influencers typically aim to influence the behavior and opinions of their followers through their posts and stories, often related to content such as fashion, beauty, fitness, travel, food, or lifestyle (Roccapriore and Pollock, 2022). In turn, they may be paid by brands to further promote certain products and/or services to their followers. To become a successful Instagram influencer, individuals must build online social communities and present themselves in a likable way to get rewarded in the form of more subscriptions, likes, and viewers (Abidin, 2016; Duffy et al., 2021).

In the world of micro-influencers, “authenticity is currency,” as those individuals with a smaller number of followers can have more frequent and genuine interactions with their followers, responding to comments and messages and sharing personal stories (Urwin, 2022). Micro-influencers promote products that are also relevant to their own interests and/or expertise. They aim to build strong relationships and communities around the content they share and “both textually and visually exhibit their personal daily lives to a large number of followers” (Chae, 2018, p. 246). They leverage SNSs to create a sense of self by interacting with followers online (Abidin, 2016; Roccapriore and Pollock, 2022). More broadly, they express different fragments of their evolving identities on social media platforms, selected for specific audiences (Abidin, 2016; Roccapriore and Pollock, 2022; Rüther et al., 2023).

### 2.2 Sample

In this article, seven Instagram micro-influencers from the Netherlands were selected as extreme cases to explore the phenomenon of online identity work (see Table 1 for the participants' characteristics). Extreme case sampling is a purposive sampling technique that allowed us to gain a deeper understanding of the dynamics of online identity work and the unique characteristics of individuals who actively engage in this process on SNSs (Eisenhardt, 1989; Patton, 1990). Specifically, the rationale behind this sampling strategy is based on three key considerations. First, Instagram micro-influencers have a significantly higher reach and visibility than the average social media user. This allowed us to explore the nuances of online identity work in the context of an audience that is beyond the reach of more typical cases. Second, the public, consistent, and active use of Instagram by micro-influencers allowed us to explore their temporal online identity work. Third, Instagram micro-influencers have high engagement metrics (e.g., likes, comments, and shares on their posts) (Duffy et al., 2021). By studying individuals with high engagement levels, we were able to gain deeper insights into the identity work strategies employed and the reactions to these strategies from an online audience. To conclude, given that Instagram micro-influencers use SNSs to create a sense of self and to perform identity work in a public and consistent way (Carter and Grover, 2015; Piszczek et al., 2016; Barros, 2018; Moser and Ashforth, 2021; Barros et al., 2023), thoroughly examining their lives provided us with an extreme context in which the process of online identity work became highly visible.

**Table 1:** Participant characteristics.

**Pseudonym**	**Gender**	**Age**	**Job title**	**Number of followers**	**Number of interviews**	**Number of posts analyzed**
Louise	F	25	Marketing employee	1,300	2	150
Jasper	M	30	Fitness coach	21k	2	1,500
Simon	M	29	Model / DJ / Photographer	5,000	1	280
Luke	M	33	Project manager	18.2k	1	500
Lisa	F	25	Marketing employee	54k	2	640
Carly	F	31	Life coach	45.2k	2	1,900
Dagmar	F	28	Marketing employee	16.8k	2	2,100

Selection criteria included the influencers' level of activity, engagement on Instagram, and their willingness to participate in the study. We aimed for diversity in terms of gender, length of time participants had been active on Instagram, and follower counts. Participants had to have between 1,000 and 100,000 followers and use Instagram consistently and actively. The nature of this population presented challenges in terms of recruitment. That is, they were considered a hard-to-reach sample, as we had difficulty getting responses to participate in this study. Therefore, two potential candidates who met our inclusion criteria were initially recruited through personal contact. The other five candidates were then recruited through snowball sampling, using a referral system through the first two micro-influencers in the sample. While the snowball sampling technique allowed us to overcome the challenges of accessing this sample, it is important to acknowledge that this method may introduce referral bias. That is, the participants were likely to refer other Instagram micro-influencers with whom they shared certain interests. In particular, Instagram micro-influencers in the health and fitness industry were overrepresented in our sample. We took this potential bias into account when interpreting the results.

In addition to referral bias, it is important to acknowledge other potential sources of bias when using Instagram micro-influencers as an extreme case. One source of bias is the influence of the influencer industry. That is, micro-influencers may have unique online identity work motivations and incentives that are more tied to brand collaboration and monetization (Banet-Weiser, 2012; Duffy and Hund, 2015; Abidin, 2016), which may differ from the motivations of more typical Instagram users. Specifically, Instagram micro-influencers are also active online to generate income through sponsorships (Duffy et al., 2021). This financial focus could also lead them to prioritize content that is more marketable, potentially influencing our findings regarding online authenticity and individuals' motivations for engaging in online identity work. Another potential source of bias is that we chose to use a single-site study to examine the dynamics of participants' online identity work. We intentionally chose to study only the participants' Instagram accounts because, on the one hand, Instagram is characterized by storytelling capabilities and a focus on sharing personal moments through mainly visual content, and on the other hand, all the participants were active on Instagram. Only a few of them were also active on TikTok, YouTube, and/or blog accounts. Therefore, findings from the Instagram platform may not be generalizable to other social media platforms, as each platform has unique characteristics and user behaviors – a topic we return to in the limitations section.

That being said, the results of this study should be interpreted in the context of these extreme cases, recognizing that they may not fully represent the more typical Instagram users. Because our focus was on gaining an in-depth and longitudinal understanding of our participants' experiences and behaviors associated with identity work, we immersed ourselves in the lives of these seven cases for whom their identity work processes are exceptionally visible in their online accounts. Although we did not strive for generalizations in these interpretations, the results may be taken to reflect on the experiences and behaviors of other individuals who are also active on SNSs. In conclusion, we respected the ethical concerns related to our (online) data collection procedure (Barros, 2018). We ensured that no private data was used in our article and that participants remained anonymous by using pseudonyms and not referring to any information (e.g., pictures) from the public accounts that could identify them. All participants provided written informed consent for their involvement in the research.

### 2.3 Data collection: a three-stage process

Data were collected through the use of complementary multi-method data, including (1) narrative interviews, (2) online autobiographical narratives of the participants' Instagram timelines, and (3) follow-up interviews. By combining these three types of data collection, we were able to take a dynamic and temporal perspective.

*Stage 1: narrative interviews:* First, we conducted narrative interviews, which were audio recorded and transcribed verbatim. The interview protocol started with the question: “Could you tell me more about your youth and how you experienced your upbringing?” Participants were then asked about the different stages of their lives, how they balanced and navigated their work, private, and online lives, the reasons for having started their Instagram accounts, and what opportunities and obstacles they encountered. By telling their life stories, the participants reflected on their identities, the sequence of different life events, and the turning points in their lives that affected their identity work over time. These interviews varied in duration, ranging from 1 h to 1 h and 45 min.

*Stage 2: Instagram timelines:* Second, the narrative interviews were complemented by an analysis of the timelines of the participants' Instagram accounts. While the narrative interviews provided us with background information about the participants' lives, they did not provide a “sufficiently faithful and detailed account of their daily practices” of their online identity work *per se* (Latzko-Toth et al., 2016, p. 205). The Instagram accounts of the participants, however, can be considered as powerful autobiographical accounts that can help to construct and share (online) self-narratives (McAdams, 1999). These timelines are well-suited to explore the dynamic elements of the participants' online identity work. The multimodal posts on these accounts included both textual (e.g., captures and hashtags) and visual/audio content (e.g., pictures and videos). We extracted the text of the posts from the accounts and compiled them into a system, along with the engagement metrics (i.e., number of tags, likes, and comments) and date of publication. This resulted in a total of 540 pages of text. Overall, the analysis of the participants' Instagram timelines resulted in an average of 965 (ranging from 150 to 2100) data points (i.e., Instagram posts) per participant.

*Stage 3: follow-up interviews:* Third, to ensure methodological rigor, follow-up interviews were conducted 2 years after the narrative interviews. Five of the participants agreed to take part. Two participants declined to participate. One participant declined the follow-up interview but did not specify a reason for his withdrawal. The other participant, who initially agreed to the follow-up interview, encountered scheduling difficulties and ultimately expressed an inability to find time for the follow-up interview. The follow-up interviews with the other five participants added to the thickness of the data, as we reflected with them on the last 2 years of their lives. During these interviews, we discussed the patterns we had uncovered in our analysis with the participants, which gave us a space to check our initial interpretations (Latzko-Toth et al., 2016). The time stamps coming from the Instagram accounts were used to enrich the follow-up interviews, as we reflected together with the participants on the changes they made to their online identities over time. These interviews, which ranged in length from 1 h to 1 h and 30 min, were also audio-recorded and transcribed verbatim.

### 2.4 Data analysis

**Figure 1:**
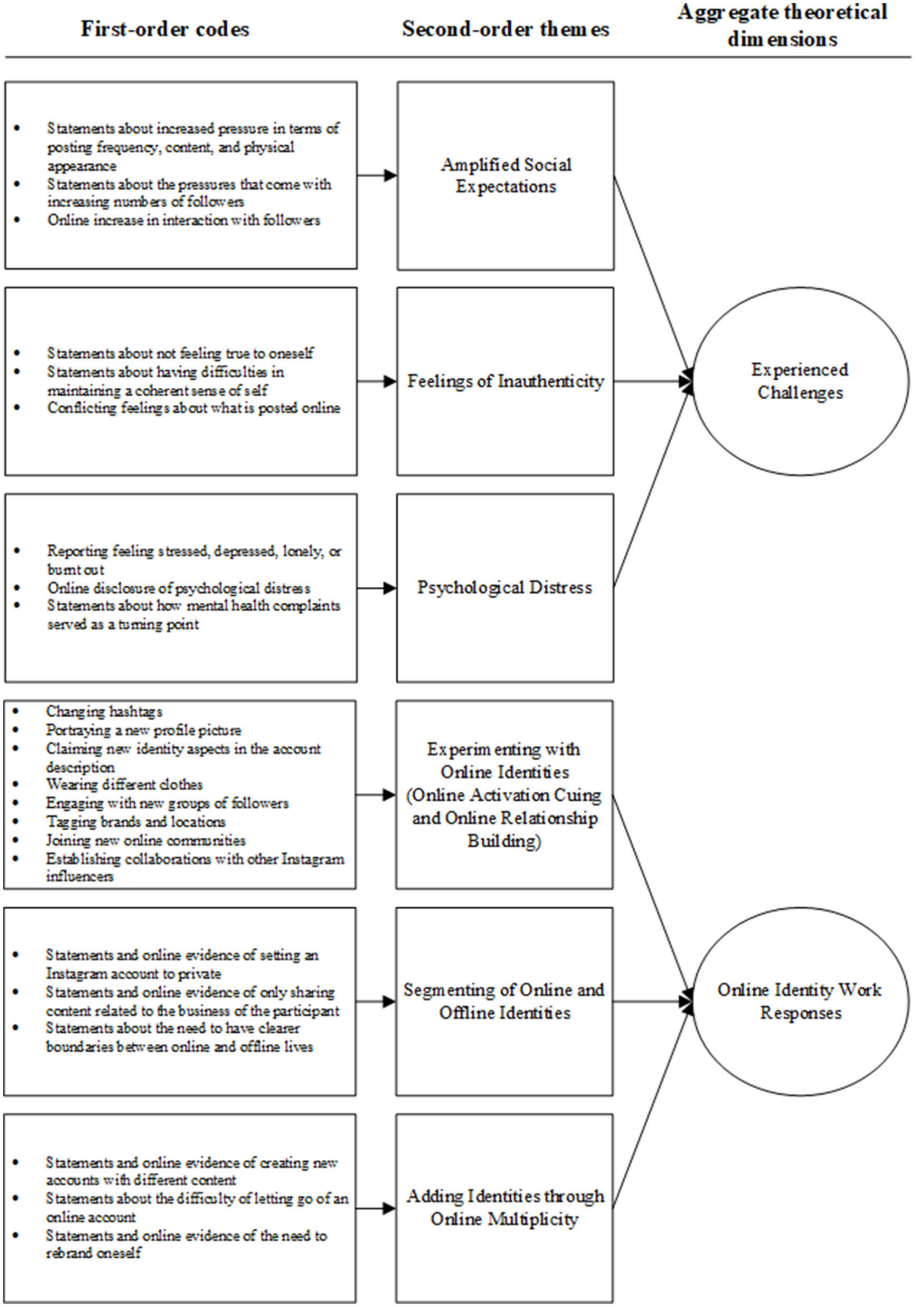
Overview of data structure.

The data was analyzed using a thematic inductive approach (Braun and Clarke, 2006), taking place in three distinct rounds. Figure 1 presents a data structure diagram that shows the overall data structure of the coding process (Corley and Gioia, 2004) and illustrates how we moved from basic first-order codes to aggregated theoretical dimensions. These coding steps are elaborated below.

First, the data was analyzed in an open and inductive manner. During this phase, we thoroughly read and re-read the narrative interviews, and went through the online textual phrases and hashtags, verbal cues, and visual and/or auditory cues coming from the participants' Instagram accounts. By going through the posts that came from the participants' Instagram accounts, we were able to discover their online identity work in a timely manner. For example, we found that for some of the participants the hashtags they used gradually changed over time (e.g., #fitgirl, #fitsociety changed to #ambitiousgirls, #entrepreneurialgirls and #girlbosses). We documented these changes and monitored how their Instagram profiles had changed over time. The first emergent findings coming from both the narrative interviews and the Instagram timelines were discussed in the research group in relation to the research question. These first ideas were then reflected upon with the participants during the follow-up interviews.

Second, first-order codes were developed to describe the main themes that we encountered in the data coming from the narrative interviews, Instagram timelines, and the follow-up interviews. These included, for example, “*changing hashtags*,” “*portraying a new profile picture*,” “*claiming new identity aspects in the account description*,” *and “wearing different clothes.”* Following our engagement with the literature and discussions in the research team, we continued our coding by grouping our first-order codes into second order themes (e.g., “*experimenting with online identities”*).

Third, and finally, the second-order themes were grouped into aggregated theoretical dimensions. This allowed us to better understand the challenges the participants faced in their online identity work and the subsequent identity work strategies they employed in response to these challenges. For example, “*experimenting with online identities,” “segmenting of online and offline identities,” and “adding identities through online multiplicity”* resulted in the aggregate dimension of “*online identity work responses.”*

In conclusion, this study was guided by a relativistic approach. This means that we acknowledge the subjectivity and contextuality of knowledge and understanding (Creswell and Poth, 2016). To ensure the rigor and trustworthiness of our research, we employed the following quality assurance methods (King and Brooks, 2016). First, efforts were made to engage with our participants over an extended period through email contact and follow-up interviews. This facilitated the development of trust and allowed for a deeper exploration of their online identity work experiences. Second, multiple data sources and methods were used to increase the robustness of our findings (i.e., narrative interviews, Instagram timelines, and follow-up interviews). Third, participants were invited to a follow-up interview to verify and validate our interpretations of their online identity work experiences. Fourth and finally, multiple authors were independently coding the data, and the authors met regularly to actively discuss the themes that emerged inductively. During these discussions, we also engaged in reflexive practices in which we acknowledged our own subjectivity and potential biases, which was key to shaping our interpretations (King and Brooks, 2016).

## 3 Results

**Figure 2:**
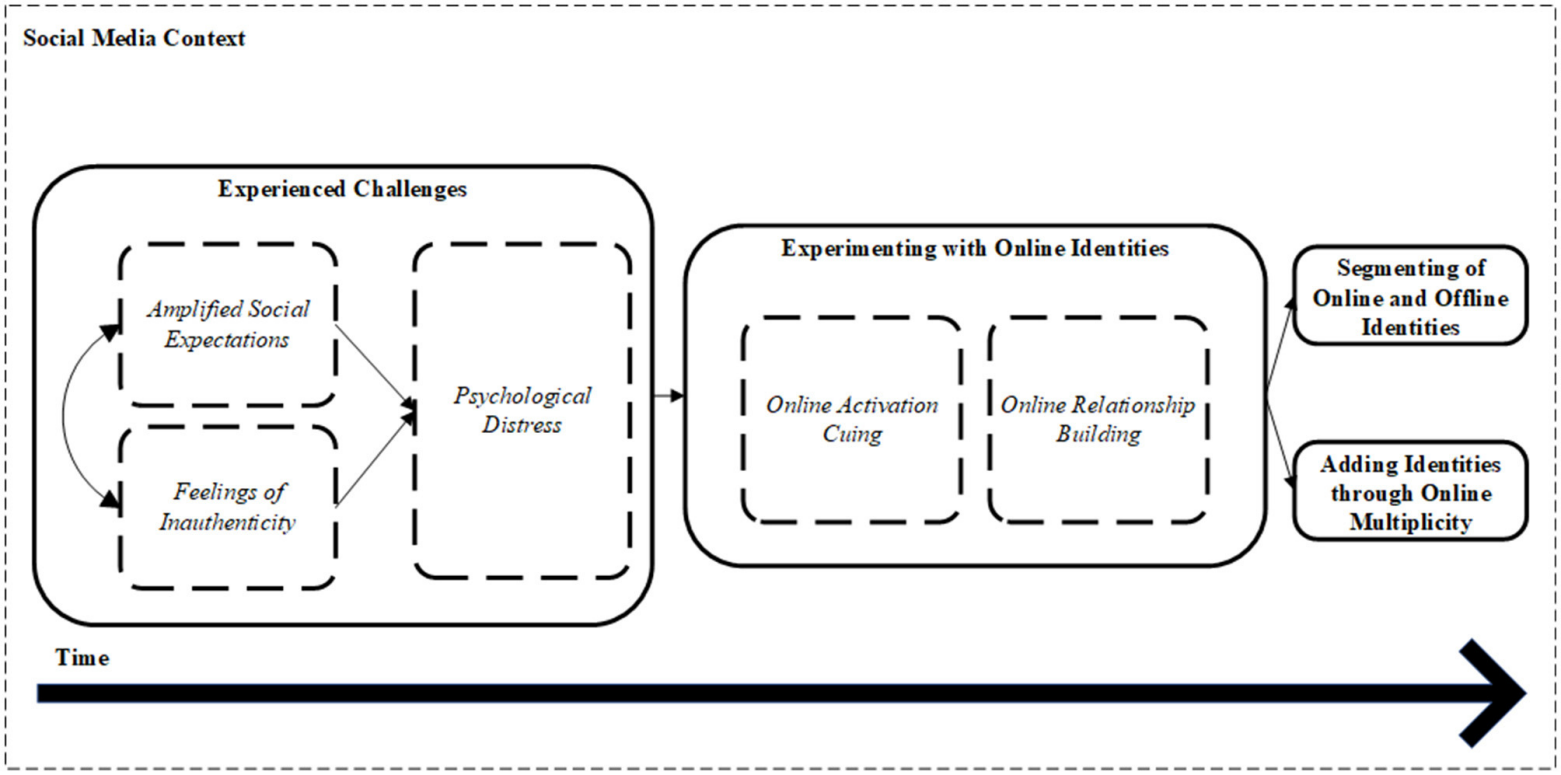
Theoretical framework of the online identity work processes.

In this section, we will present the themes and subthemes that emerged from our data analysis, with evidence coming from the narrative interviews, the timelines of the participants' Instagram accounts, and the follow-up interviews. We identified three main themes that highlight the challenges that the Instagram micro-influencers encountered online: (1) amplified social expectations, (2) feelings of inauthenticity, and, as a result thereof, (3) psychological distress. We found that these challenges were viewed by the participants as catalysts for their online identity work processes. More specifically, we identified three key online identity work strategies that were employed in response over time: (1) experimenting with their online identities, (2) segmenting of their online and offline identities, and (3) adding identities through online multiplicity. Figure 2 presents our theoretical framework which forms the basis for our results section.

### 3.1 Experienced challenges

The theme “experienced challenges” is defined by the challenges that participants experienced online. In the interviews, participants shared how they experienced the amplification of social expectations, their feelings of inauthenticity, and, as a result thereof, psychological distress.

#### 3.1.1 Amplified social expectations

In the analysis, it became clear that as the participants engaged more with social media, in due course, all of them started to face challenges stemming from the increased social expectations they experienced. They explained that when they first started using social media, they did it mostly for fun. Over time, however, they described how their growing number of followers led them to experience increased pressure to meet the perceived expectations in terms of posting content more frequently and the type of content they shared online. Louise, for example, described her experience of a significant increase in the number of followers she had, which led to difficulties in meeting the increased social expectations that came with it:

“*Within 3 months, I had like 500 new followers you know? And that also gradually increased the pressure, since you need to deliver content. And then you think, oh, now I have 160 likes and then I had 102… What is wrong? Didn't I use enough hashtags? Does my face look weird? Is my hair okay? I don't know… What do they think of me?” (Louise, narrative interview, 08/11/20)*

Similarly, Lisa mentioned that she had gained 10,000 additional followers in a short period of time and explained how she experienced psychological difficulties in balancing the expectations that these followers created. She described how she began to feel severe pressure to perform on her online account, driven by the expectations she perceived for her to produce content production on a regular basis, such as a weekly video or frequent posts.

“*Yeah, I also feel a lot of, uh, pressure to perform. Because I often feel like something is expected of me, like a video every week or content every few days. I don't know, last month I got 10,000 new followers. Yeah, it really shot up, but it also comes with the fact that I get a lot of private messages. I really want to answer them all, I really do, and I want to take my time. But I get 50 new messages every day and they're really long, because they're all people with their own stories who want to lose weight. I think it's really nice and sweet that they want to share that with me and get my opinion or whatever, but I just don't have the time. I don't have the time to respond in detail and it stresses me out, because I want to respond and I want to help these people in my own way (speaks quickly) and I find it very challenging to, yeah, combine and balance that.” (Lisa, narrative interview, 08/01/21)*

Participants explained how online social expectations were not only about responding and replying to messages, but also about their physical appearance and the content they presented on their timelines. These expectations came from both their followers and potential business relationships:

“*In the world of modeling, there is an increasing focus on, alright, who are you? That becomes more important. So, when I have a modeling job abroad, then I also need to film things around it, show something, those kinds of things. And that is what they find interesting online.” (Simon, narrative interview, 08/12/20)*

“*It is difficult. Because at a certain moment in time, I made that decision to go into the fitness world. And that means that I always must be fit, I always need to look good.” (Jasper, narrative interview, 01/12/20)*

“*And then Instagram came along, so you start looking at it and you see all those…, you know, then you start taking those protein shakes. I started exercising even more. Um, then I started sharing about it on my Instagram. Then you enter that world of fit girls. And then, it just gets worse, it just gets more. I was just trying to achieve a perfect version of myself. It was never good enough. I wanted a big bum, but I also wanted to be super skinny. Yeah, when I look back now, I really think I had such a blindfold on! Every time I looked in the mirror, it was not good enough. And when I look at those pictures now, I think: Jesus, why wasn't I good enough, you know?” (Dagmar, narrative interview, 19/03/21)*

In general, the participants in the study discussed the gradual increase in social expectations they encountered over time and their efforts to meet these expectations, especially those coming from their followers. A detailed analysis of Carly's Instagram account revealed a gradual change in her content strategy. Initially, she shared only photos of her visits to the gym and her food choices. Over time, however, her content expanded to include detailed descriptions and videos of her workout routines, as well as before-and-after photos that showcased her physical transformation. Concurrent with this shift in content, we saw a significant increase in their follower count and engagement levels. Table 2 provides a visual representation of this growth, showing a significant increase in the number of likes and comments she received on her posts over a period of 4 months. Carly liked and replied to all of her followers' comments. This data example highlights Carly's increased commitment to sharing her personal progress online and engaging with her followers, reflecting her efforts to meet increasing social expectations online.

**Table 2:** Exemplary posts of Carly over time.

**Time →**	**→**
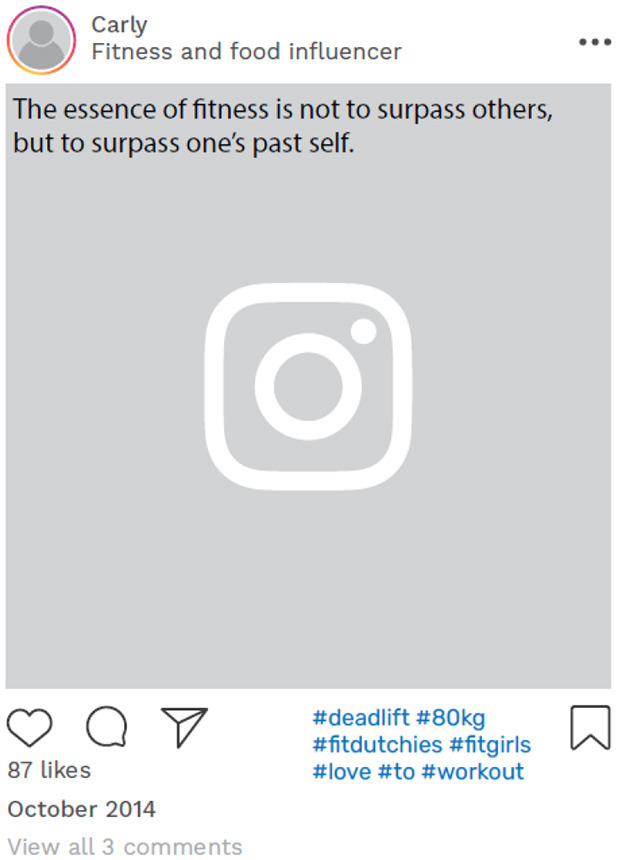	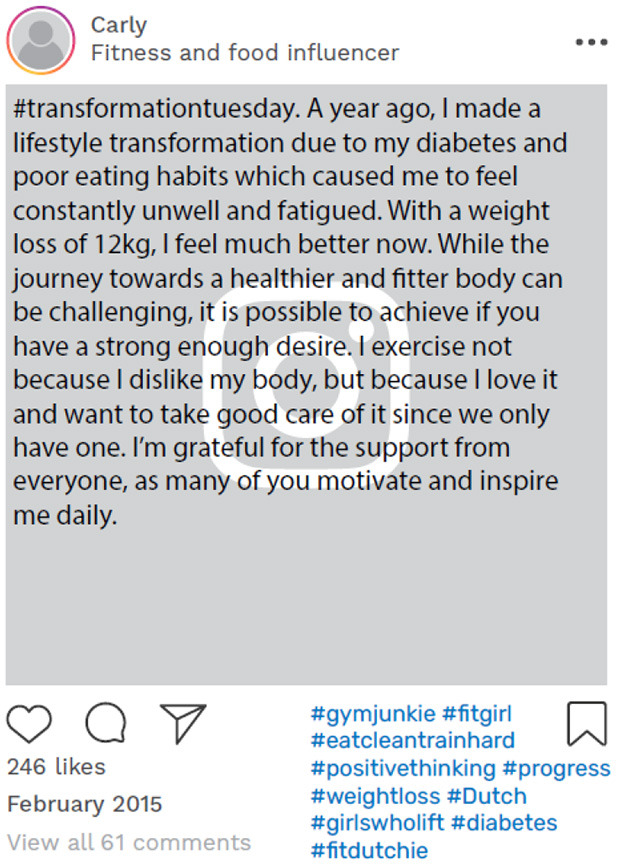

Instagram posts designed and reproduced from s.salvador via Freepik (https://www.freepik.com/free-psd/instagram-post-template_5263426.htm#&position=1&from_view=author&uuid=2410d69f-1272-4628-a0b1-836d31b3bb20).

#### 3.1.2 Feelings of inauthenticity

In describing their experiences with social media over time, six of the participants also shared how they gradually experienced feelings of inauthenticity. These participants articulated the challenge of presenting a consistent and true self online. In her narrative interview, for example, Carly described how her online identity had evolved into an external image that she felt compelled to maintain over time:

“*Well, I have maintained that (online) image for such a while, that I maybe think that I still am that person. But yes, I now start to doubt a bit. I am a bit stuck in the middle. On the one hand, I really like it, and on the other hand, I question whether that is still the person that I am? You know, you create a certain identity for yourself or so. (…) You think, you must be like that. I think that is it. That I must be like that. And I also somehow don't want to let go of it.” (Carly, narrative interview, 26/02/21)*

Strikingly, Dagmar, Louise, Jasper, and Lisa shared similar experiences of feeling increasingly inauthentic as they tried to meet their followers' expectations. They described the difficulty of maintaining a coherent sense of self and presenting a consistent identity across online and offline contexts. Simon, for example, explained:

“*The most difficult of this life is that like a year ago I was thinking like, okay, but what is really your identity you know? Yes, you are seen as this social guy, and you are often at many places, you are always surrounded with a lot of people, you are a social dude. But yeah, what do you really want yourself? It is often like, you go from one thing to the next, and it all just continues. But then you sometimes stand still, and you think, who am I now and where do I want to be in 5 years?” (Simon, narrative interview, 08/12/20)*

While the participants acknowledged the challenges of maintaining a consistent self across online and offline environments, our analysis of the Instagram accounts revealed that they persistently posted images and videos that largely conformed to their followers' expectations. For example, Louise, who struggled with an eating disorder in her daily life, maintained her online identity as a food blogger. She explained that she had difficulty sharing her negative experiences on social media:

“*And to the outside world, I did this by starting my online blog, making my Instagram public. Only smiling, eating, whereas I have an eating disorder. It is not right. But I am keeping up appearances because I don't want other people to know that I am not doing well. That is also why I keep work and private strictly separate. So even if I am sitting here crying on the couch, I still must provide content. So that is very conflicting. Because then I posted a photo of me smiling, and you don't know what's going on behind it. And people think I am really that fun.” (Louise, narrative interview, 08/11/20)*

**Figure 3:**
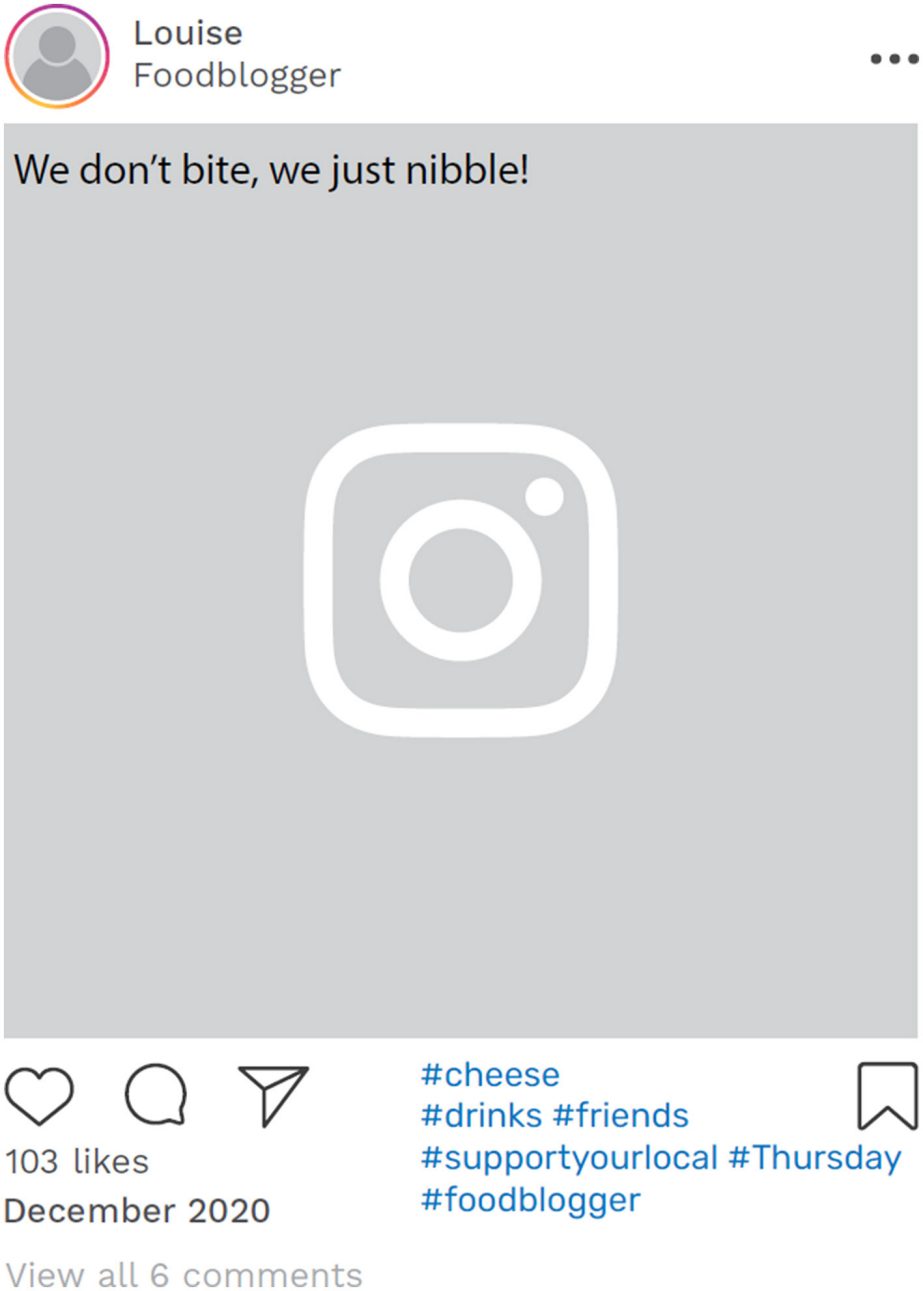
Example of online data taken from Louise's Instagram account. Instagram posts designed and reproduced from s.salvador via Freepik (https://www.freepik.com/free-psd/instagram-post-template_5263426.htm#&position=1&from_view=author&uuid=2410d69f-1272-4628-a0b1-836d31b3bb20).

The challenge of presenting a consistent identity across different environments, both online and offline, was further highlighted by our analysis of Louise's Instagram timeline. A month after the narrative interview, in December 2020, her account still presented her as a “food blogger,” with predominantly content centered around happiness, food, and wine (Figure 3).

In general, six of the cases revealed an increasing sense of inauthenticity, even though they attempted to maintain a consistent self across their online and offline environments. Despite the participants' struggles, however, our analysis of their Instagram accounts revealed their unwavering commitment to meeting the expectations of their online followers. As a result, many participants described how they experienced increasing psychological distress over time.

#### 3.1.3 Psychological distress

All participants, except for Luuk, expressed how they experienced psychological distress over time. They explained how the increased social expectations and feelings of inauthenticity threatened their wellbeing, leading to feelings of loneliness, stress symptoms, depressive complaints, and burnout complaints. The following example from Lisa shows how she experienced stress due to the conflict between her online (work) life and her personal life:

“*Well, I am just very stressed. I really want to have an evening to myself, but it just never seems to happen, it never does. And I'm someone who experiences a lot of stress very quickly, because I want to - I only have no stress left when everything on my list is done, but it never happens! It doesn't happen at work, and it doesn't happen in my personal life. Yes, yes, I have a lot of stress. And then I just don't know what to do first, I'm just not relaxed, you know. I just don't know what to do first.” (Lisa, narrative interview, 08/01/21)*

Like Lisa, Jasper shared how the challenges he faced online led him to experience psychological distress. He explained how he initially resisted the idea of experiencing burnout complaints and continued to try to live up to the expectations of others he felt, even though his body was signaling the need for rest. Ultimately, his decision to work with a psychologist was a turning point in addressing the challenges he was facing:

“*What your body does then, your body starts to sort of put things into perspective, and that's what my body started to do. So, suddenly it went like, yeah, you've had to perform for so long, it's time to take a break. But I didn't deal with it, so I could have sat at home and worked on it, but I never did because I personally didn't really believe in it. I didn't believe in burnout at all, so I just kept going. But what I started doing is working with a psychologist to process things and understand where things are coming from.” (Jasper, follow-up interview, 04/11/22)*

**Figure 4:**
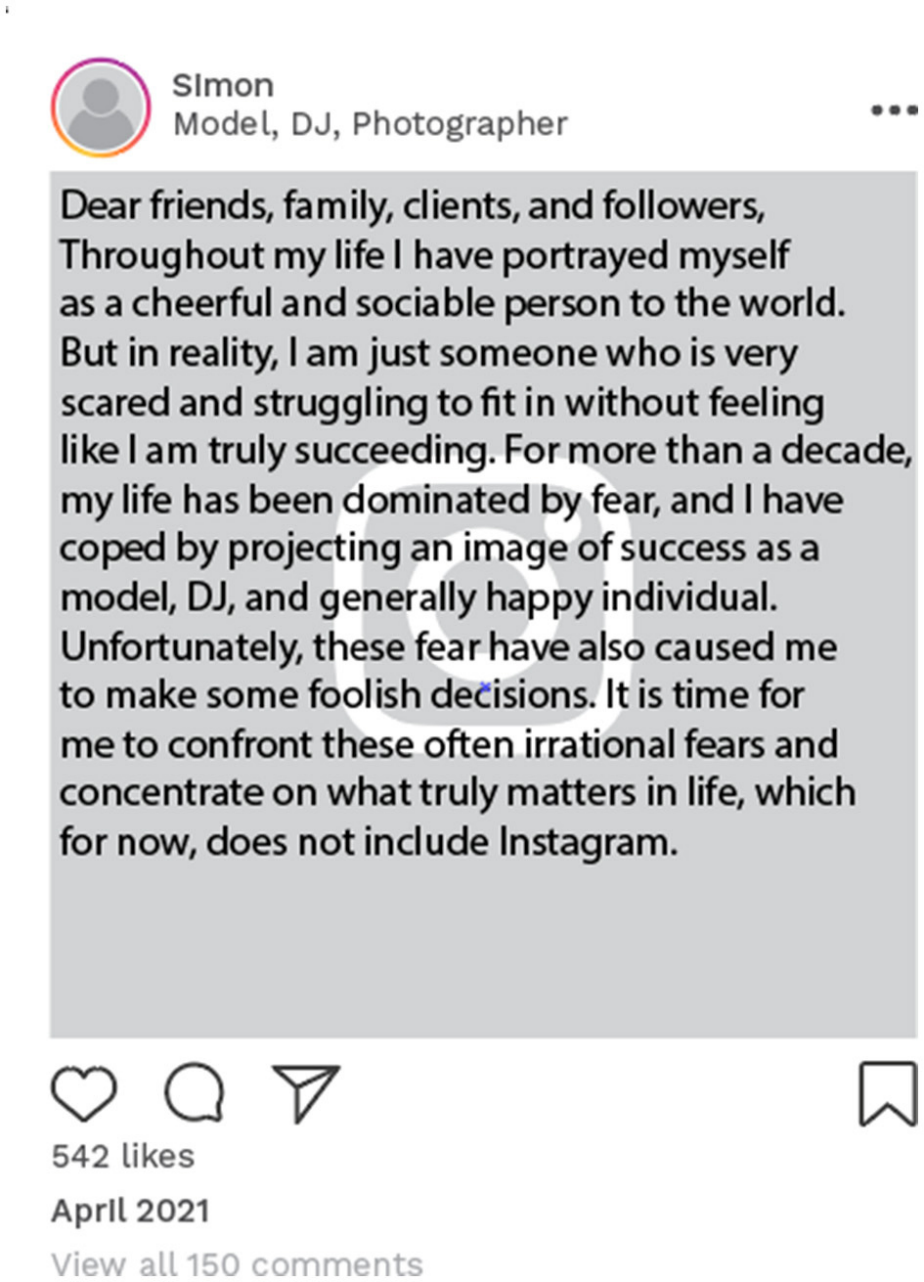
Example of online data taken from Simon's Instagram account. Instagram posts designed and reproduced from s.salvador via Freepik (https://www.freepik.com/free-psd/instagram-post-template_5263426.htm#&position=1&from_view=author&uuid=2410d69f-1272-4628-a0b1-836d31b3bb20).

**Figure 5:**
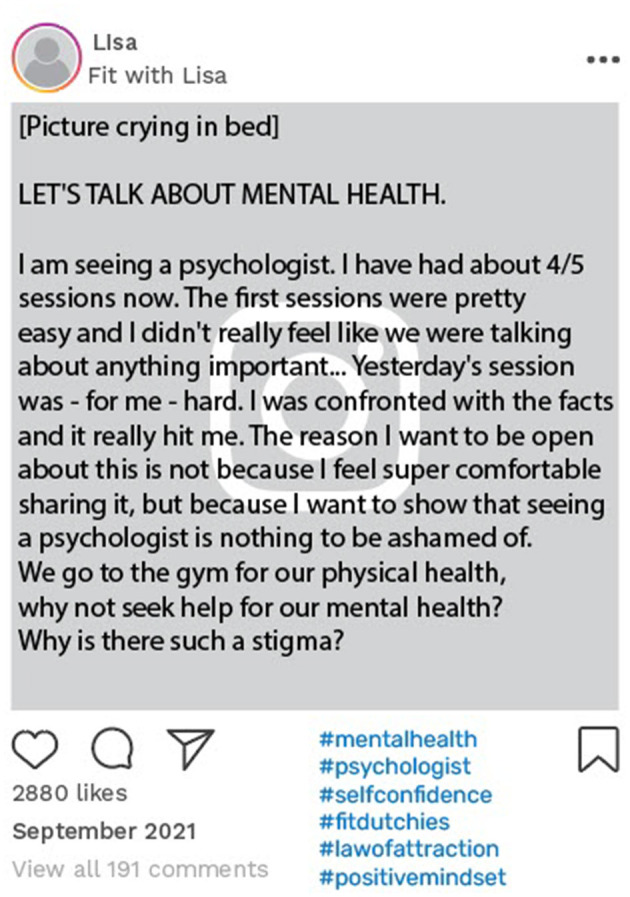
Example of online data taken from Lisa's Instagram account. Instagram posts designed and reproduced from s.salvador via Freepik (https://www.freepik.com/free-psd/instagram-post-template_5263426.htm#&position=1&from_view=author&uuid=2410d69f-1272-4628-a0b1-836d31b3bb20).

Carly, Lisa, and Simon had been open with their followers about their feelings of psychological distress. An example of this is Simon's online disclosure of his struggle with a major depression. This was posted 4 months after his narrative interview, in which he had already discussed the challenges he experienced of maintaining a consistent identity. In this particular social media post (Figure 4), Simon openly shared his personal struggles and engaged with his followers. Similarly, Lisa disclosed that she was seeing a psychologist and told her followers that she wanted to be open about her negative health experiences (Figure 5).

In our data analysis, it became evident that the participants struggled with amplified social expectations and feelings of inauthenticity online, which, in turn, contributed to their experiences of psychological distress. This psychological distress emerged as a catalyst for change:

Louise's breakdown:

“*I am not going to maintain doing this. I notice that I have reached a point and that was only recently, because I started to break down my walls. But that was when I was with my parents and said: ‘I can't take this anymore' and I said: ‘within 3 months, I won't be here anymore.' Either I fall out mentally within 3 months, or physically. Something must happen right now. Yeah… that.” (Louise, narrative interview, 08/11/20)*

Carly's turning point:

“*At one point I had so many followers that it became overwhelming, and at one point I completely collapsed. It happened during a vacation. I thought things were going well, but when you're always on, you have a constant level of stress, very high stress. So, we went on vacation, and I thought, ‘hey, maybe I should try to do nothing for 5 days,' because we only went to my in-laws for 5 days. But I couldn't even do that. I still wanted to answer messages. Then at one point we went out to dinner, and I just couldn't enjoy it. I realized that I still couldn't really enjoy anything. So, at one point, for no apparent reason, I started crying, and I couldn't stop. I went back to the hotel for a while. I cried all night and wrote down everything I was feeling. It was mostly the pressure that I felt that I always had to be there for everyone. That was the turning point.” (Carly, narrative interview, 26/02/21)*

Dagmar's reevaluation:

“*Well, I think I was just working less anyway, but not in a way where I was like: “Oh, that's great, now I can be really productive with other things.” No, I was just lying on the couch, and I was already stressed about the smallest things. So, I really realized, okay, this is not making me happy anymore. I also realized that I wasn't finding happiness in the requests that I was getting from followers or the constant need to post. I was kind of done with that for a while. So, yeah, that's when I made the decision to throw it overboard.” (Dagmar, follow-up interview, 08/11/22)*

These examples highlight the interplay between the amplified social expectations, feelings of inauthenticity, and, as a result thereof, psychological distress. In turn, psychological distress triggered online identity work responses.

### 3.2 Online identity work responses

The theme of “online identity work responses” is defined by the changes that participants made to their online identities in response to the challenges they faced. The data revealed three different online identity work strategies that were employed in response to the experienced challenges: (1) experimenting with online identities, (2) segmenting of online and offline identities, and (3) adding identities through online multiplicity.

#### 3.2.1 Experimenting with online identities

The first online identity work strategy we discovered, particularly when analyzing the participants' Instagram accounts, was labeled “experimenting with online identities” and was characterized by a process of experimenting with different identity work tactics online to explore new identities. Our analysis revealed two categories of identity work tactics that the participants used for experimentation: online activation cuing and online relationship building. To illustrate these two tactics of online identity work, we purposefully selected two participants who provided us with a clear temporal example of experimenting with online identities, which we have visualized in Table 3.

**Table 3:** Exemplary posts of the online identity work strategy “experimenting with online identities” over time.

**Time**	**→**	**→**	**→**
**Online activation cuing** An online identity work tactic used to cue new online identities. It involves using cues and signals such as clothing, hashtags, profile pictures and account descriptions on online accounts to emphasize certain parts of one's online identity.	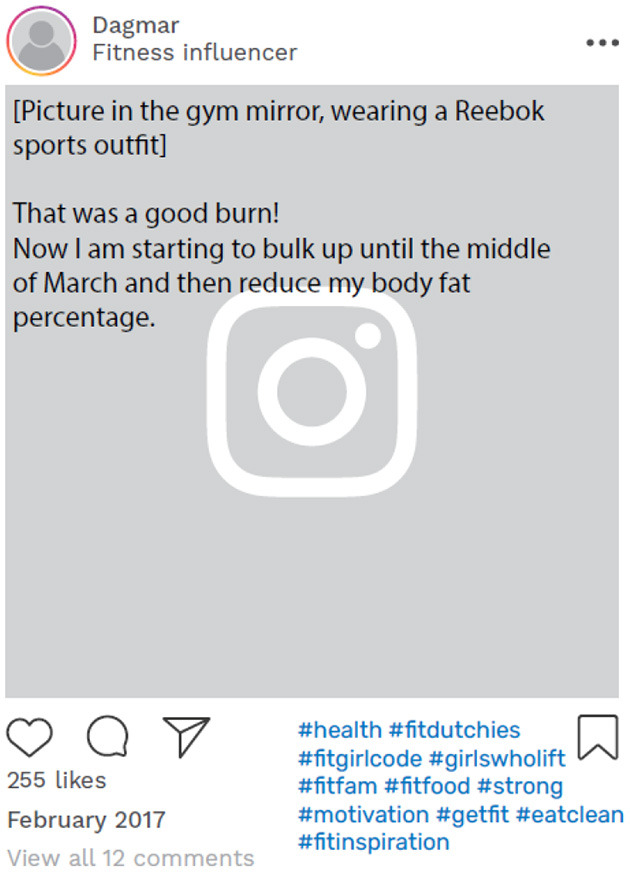	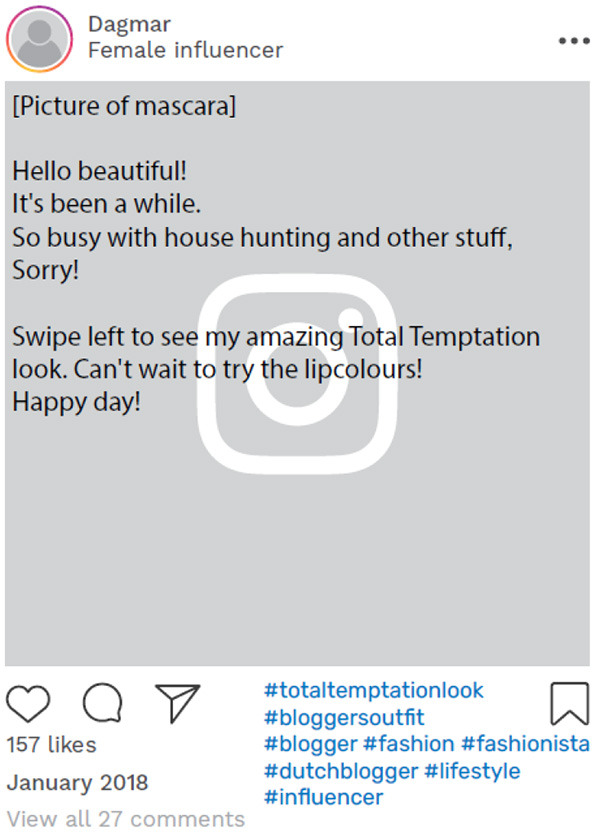	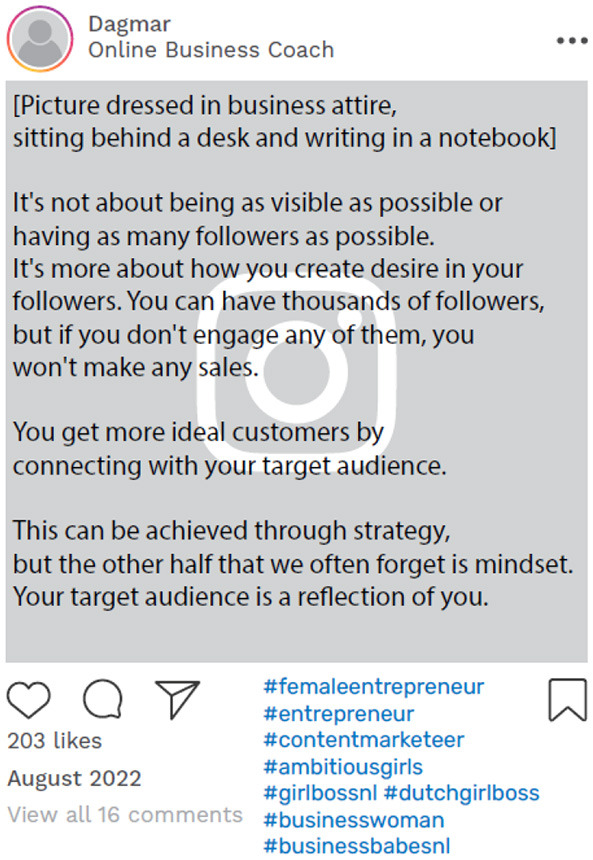
	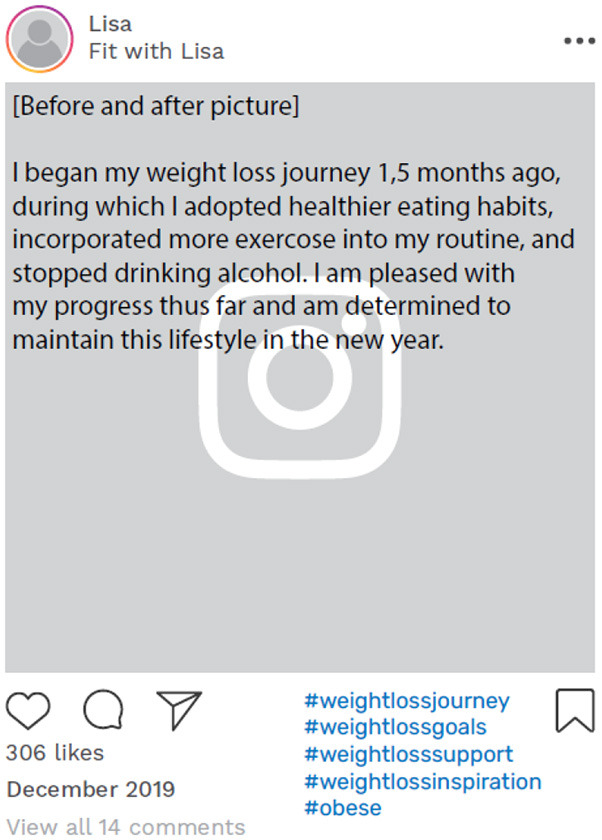	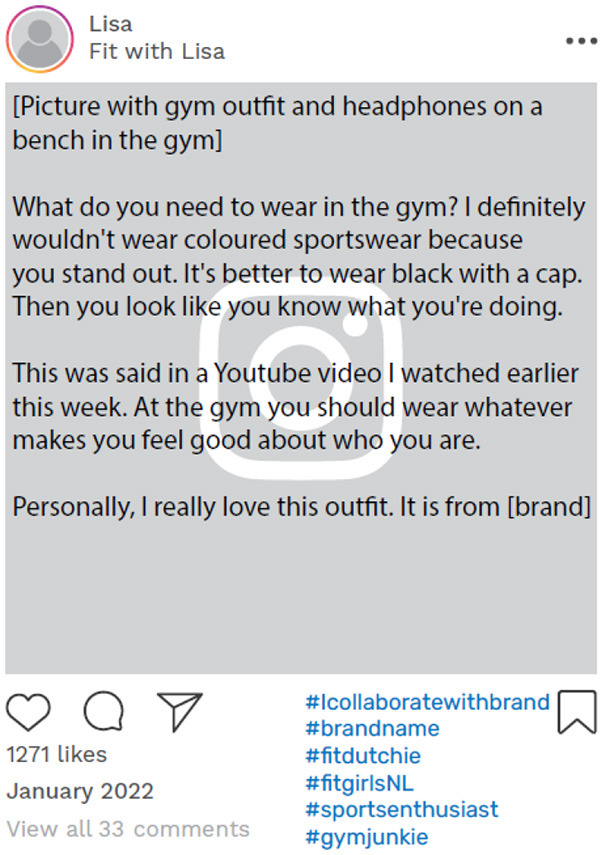	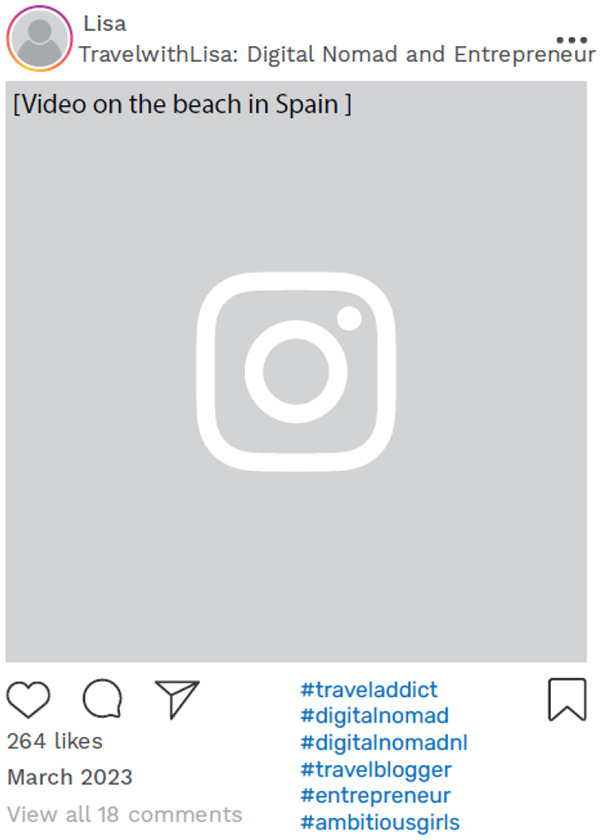
**Online relationship building** An online identity work tactic that involves creating or strengthening online relationships with others whose online presence also aligns with a desired online identity. This tactic includes joining new online communities, engaging with new groups of followers, and tagging brands and locations.	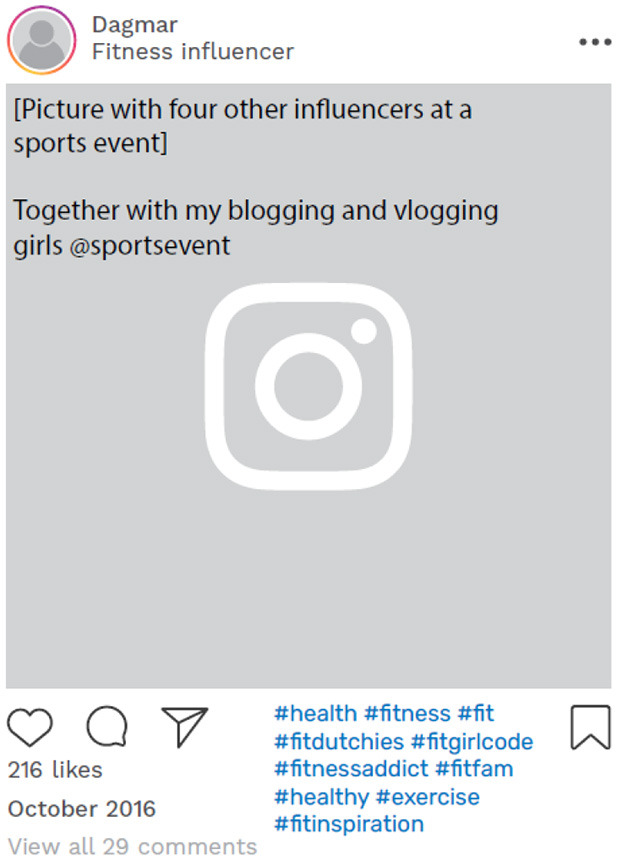	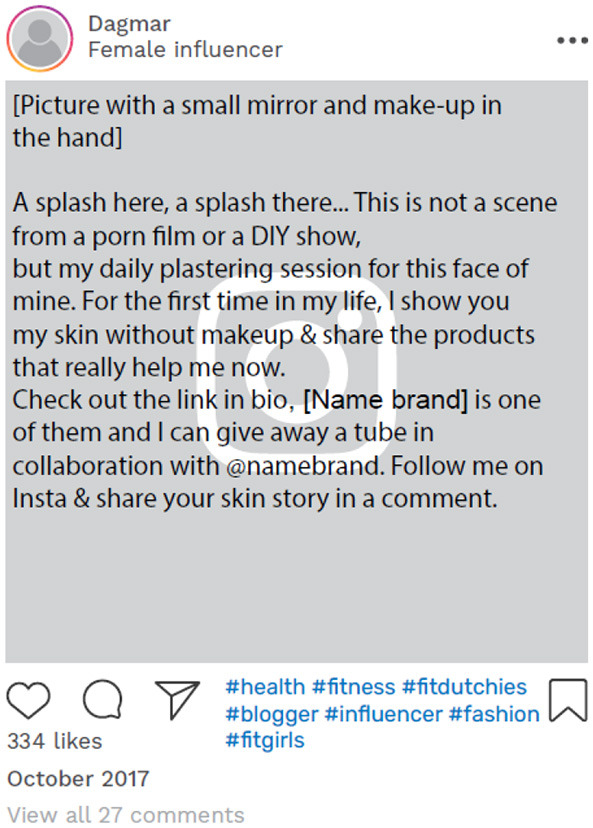	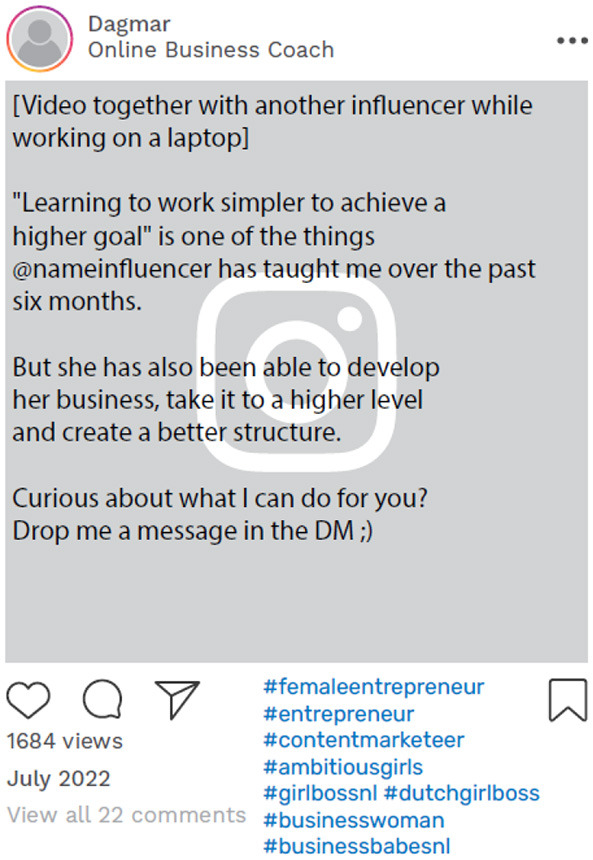
	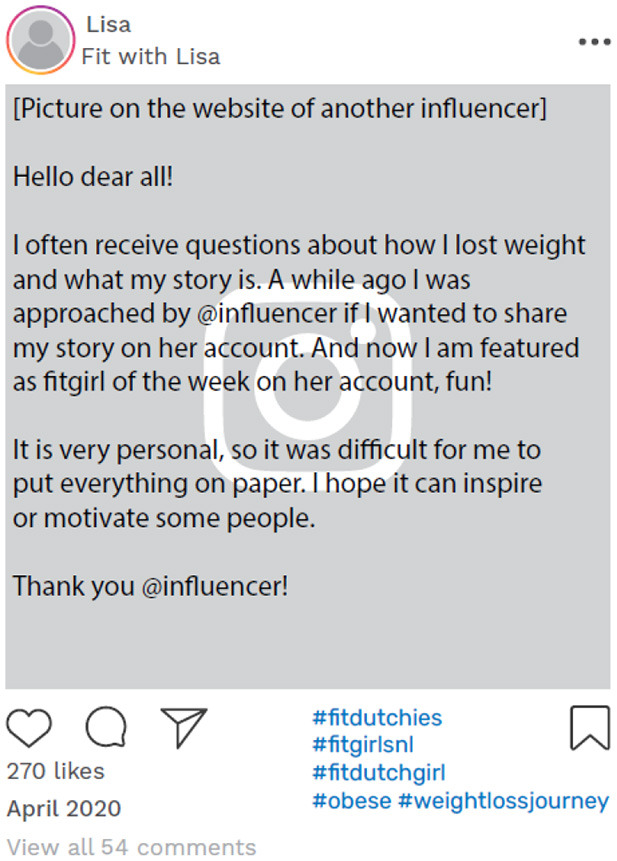	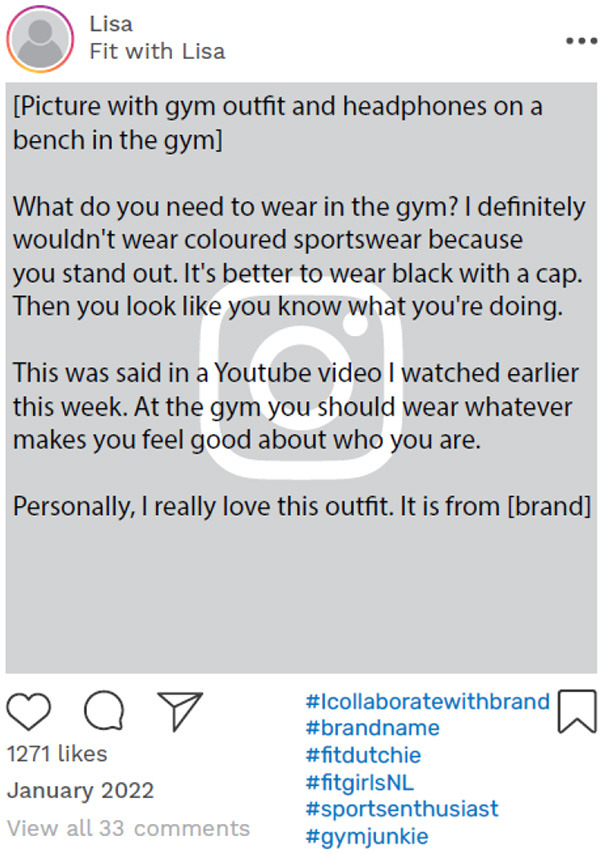	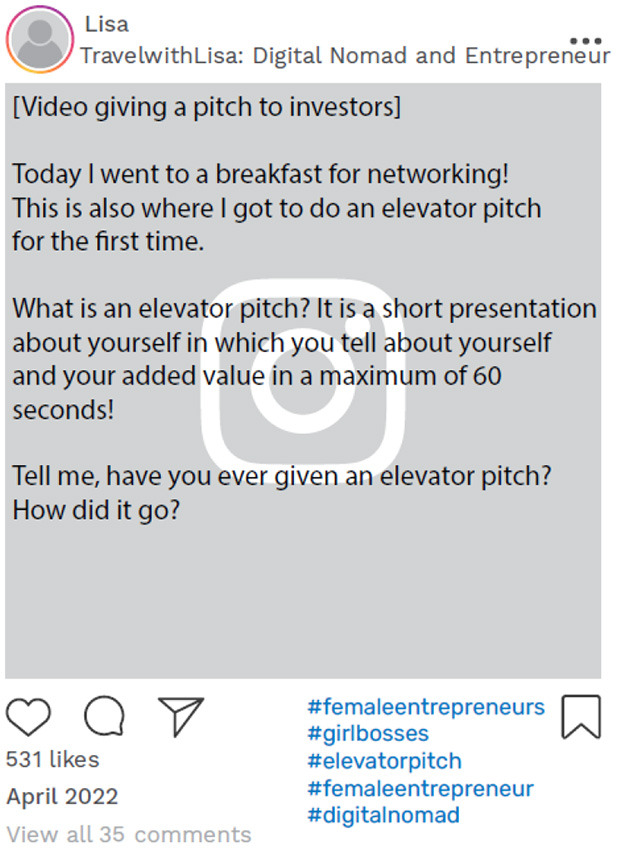

Instagram posts designed and reproduced from s.salvador via Freepik (https://www.freepik.com/free-psd/instagram-post-template_5263426.htm#&position=1&from_view=author&uuid=2410d69f-1272-4628-a0b1-836d31b3bb20).

##### 3.2.1.1 Online activation cuing

The first category of tactics, the *online activation cuing* tactics, includes how participants experimented with and began to modify their online identities by intentionally using cues and signals to accentuate different facets of their online identities. This involves a variety of actions, including the posting of content that highlights new interests, using specific hashtags, changing profile descriptions and profile pictures, and selecting clothing for online photos. For example, Carly changed her profile description from “Fitness and Food Influencer” to “Manifestation Expert” and shifted her content focus from fitness workouts and food to entrepreneurship and positive thinking. Around 2021, she began to include new hashtags such as #highlysensitiveperson and #lawofattraction in her posts, while gradually decreasing her use of fitness-related cues (e.g., #fitdutchie). Concurrent with these changes, Carly transitioned from predominantly wearing workout clothes to more smart casual outfits. Carly's follow-up interview revealed her sense of relief stemming from experimenting with these online activation cuing tactics and her perception that she was making more progress toward her authenticity online:

“*I started to feel very relieved when I started mindset coaching and making changes to my account. I was on my way to becoming more of my authentic self.” (Carly, follow-up interview, 08/12/22)*

Another participant, Dagmar, also shifted her content away from fitness and body transformation to share more about her daily life, particularly in relation to fashion and female struggles. She also started using hashtags that were more related to fashion and blogging (Table 3).

##### 3.2.1.2 Online relationship building

The second category of identity work tactics, which we labeled *online relationship building*, refers to the actions that the participants took to build relationships that were more aligned with their personal interests. This involved the deliberate creation of online connections, including followers, fellow Instagram influencers, and sponsors. Within this category of tactics, participants engaged in actions such as liking and providing comments on their followers' responses, tagging brands and locations, using hashtags to join specific online communities, and establishing collaborations with other Instagram influencers. In this way, participants established new online business and personal relationships. Lisa described:

“*You must present yourself online to get collaborations. Right now, I am working on my second book and then I am going to set up an online community. I collaborated on my book with another influencer who is a dietician, so I want that dietician in the online community. People can then join and receive information from someone who has actual knowledge on the topic. They can do live workouts, and they can also ask questions about sports. And I will be there for motivation.” (Lisa, follow-up interview, 08/12/22)*

**Figure 6:**
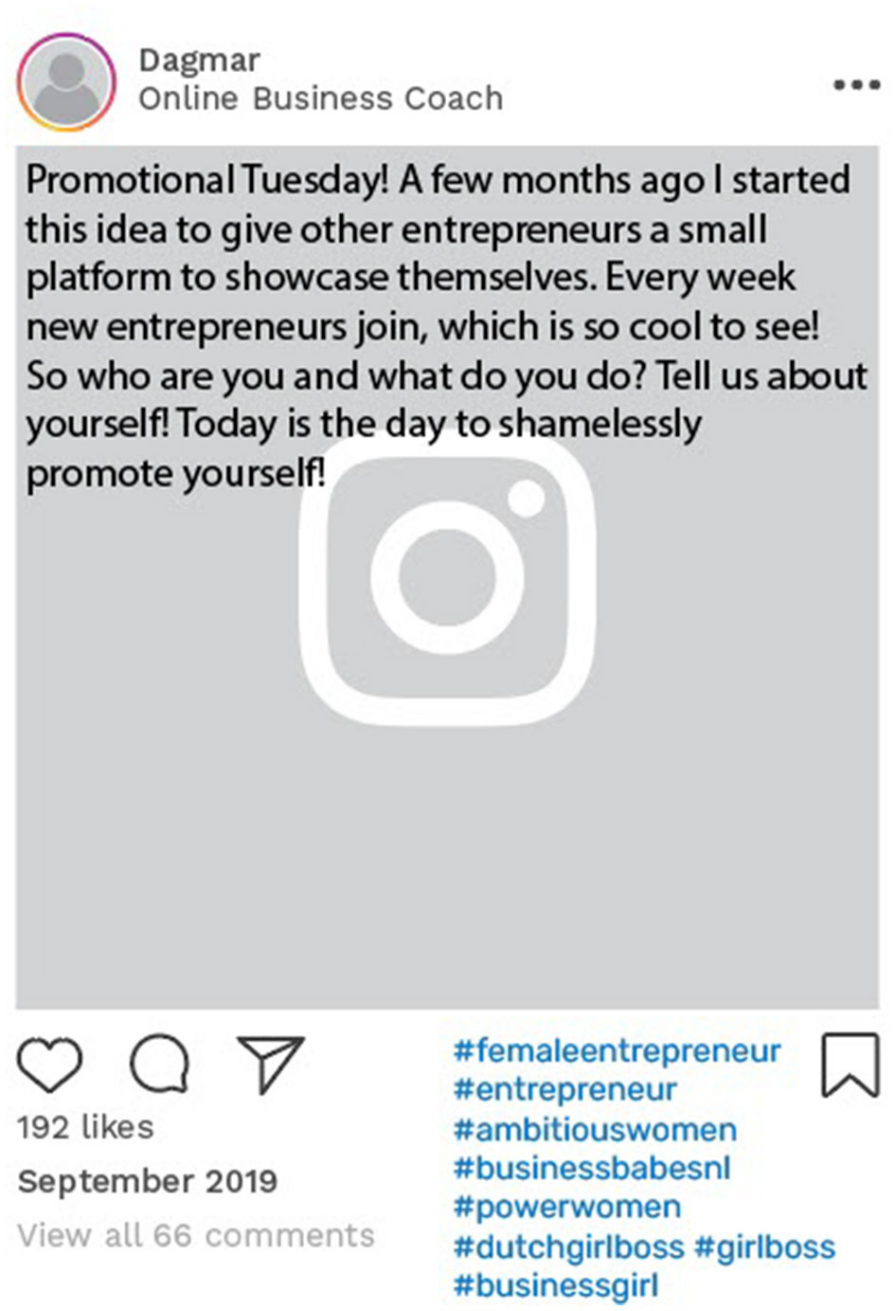
Example of online data taken from Dagmar's Instagram account. Instagram posts designed and reproduced from s.salvador via Freepik (https://www.freepik.com/free-psd/instagram-post-template_5263426.htm#&position=1&from_view=author&uuid=2410d69f-1272-4628-a0b1-836d31b3bb20).

Table 3 illustrates the evolving patterns of Carly and Dagmar's online relationship building that better aligned with their desired online identities, that is, being a health and manifestation coach for Carly and being a female entrepreneur for Dagmar. Carly, for example, immersed herself in online communities that reflected her newfound identity, incorporating hashtags such as #ambitiousgirls, #entrepreneurialgirls, and #girlbosses. In 2019, Dagmar underwent an online identity transformation by changing her account description to “Online Business Coach.” She also initiated a weekly engagement ritual with her followers, dubbed “Promotional Thursday,” where she fostered connections with her followers and encouraged them to tag other entrepreneurs (Figure 6).

Overall, our data analysis revealed that the participants initially employed experimentation tactics, including online activation cuing and online relationship building, to change their social media accounts and explore new desired online identities. Their Instagram accounts allowed them to access new online communities and monitor post analytics, thereby receiving feedback on the changes they made to their accounts and, hence, their online identities.

However, after a period of experimentation with their online identities, our analysis revealed that participants subsequently adopted one of two primary online identity work strategies to further cope with the challenges they faced: (3.2.2.) “segmenting of online and offline identities” and (3.2.3.) “adding identities through online multiplicity.”

#### 3.2.2 Segmenting of online and offline identities

In our analysis, we labeled one of the follow-up pathways chosen after the phase of experimentation as “segmenting of online and offline identities.” This strategy was employed by Luke, Simon, Jasper, and Louise, who, over the course of 2 years, underwent a process of further segmentation of their online and offline identities. This implies that initially, these participants experimented with online activation cues and online relationship building to gradually change their online identities. Over time, however, our analysis showed that they established clearer boundaries between their online and offline identities in response to the challenges they faced. Analysis of Louise's Instagram account revealed that her account had been set to private since 2022. During our retrospective follow-up interview, she explained:

“*Yes, I now fully let go. I put my profile on private, and then I thought: ‘I will just call it quits.' Also, the strategy that I used to have like: first eating, then a picture of myself, then a picture of eating, picture of myself, I just let go of that. And I really thought: ‘why do I even edit my pictures?”' (Louise, follow-up interview, 30/09/22)*

Similarly, Luke did not share any aspects of his personal life on his Instagram account and only shared posts related to hockey coaching. His posts did not include pictures of himself or stories about who he is. His posts mainly showed different hockey techniques being performed by other people on his team. Simon's account also began to only show pictures of his modeling, photography, and DJ activities. He stopped sharing other aspects of his personal identity online.

#### 3.2.3 Adding identities through online multiplicity

Another follow-up pathway that some participants took over time we labeled “Adding Identities through Online Multiplicity.” Paradoxically, the participants who took this pathway described feeling inauthentic in their original online accounts, while at the same time struggling with the reluctance to let go of those accounts. Participants described how, over time, their online identities as displayed on their initial accounts became firmly established, negotiated, and validated by a large group of followers. However, they also recognized that the social expectations imposed by these followers further complicated their decision to let go of their established online identities. This strategy was associated with Carly, Dagmar, and Lisa, who decided to create multiple online identities across different Instagram accounts. Notably, Instagram facilitated a seamless transition between these different accounts by allowing the participants to switch between multiple accounts without having to log out and then log in again for each account separately.

Carly, for example, expressed her doubts about whether her online identity as a fitness influencer really reflected who she was, even though she had maintained it for quite some time. At the same time, she struggled with the idea of letting it go as it had become an integral part of who she was online. After a period of experimentation with her old account, Carly started a new social media account, this time, focused solely on mindset and spirituality. She found that this new account gave her a “clean slate” and allowed her to connect with like-minded individuals. Consequently, she started to feel more authentic again in what she presented online. In her follow-up interview, she described this transition and emphasized her newfound sense of authenticity online:

“*And then I started a new account. And on that one I just posted about mindset and spirituality. And my old account, the one with 45,000 followers, is just kind of stagnant now. Yes, I still post Insta stories, but not as much as I used to. And I noticed that with the new account, I don't know, it just gave me a sort of clean slate. It was completely new, and I could really be myself on there for once. And that might sound strange, because you could do that on your old account too, of course. But I don't know, there were all these people on there who just wanted to see fitness stuff all the time. And yeah, I just didn't feel like doing that anymore.” (Carly, follow-up interview, 08/12/22)*

When we asked her about the challenges of letting go of her old account, Carly mentioned the persistent urge to maintain it, especially influenced by her considerable number of followers:

“*It had been in my head for a while to just close the account and create a new one. But every time you just don't do it. Yeah, you still think… Even when you talk to people about it, they always say: ‘Yeah, but you've got so many followers, you're not just going to give that up, are you?' And then you start thinking again, oh yeah, maybe I shouldn't do it yet.” (Carly, follow-up interview, 08/12/22)*

Dagmar faced a similar dilemma when she decided to change her online account. She explained that it felt like she was throwing away a successful formula that had been well-received by her followers over a long period of time:

“*I made the decision to throw everything overboard, and to just focus on one-on-one customers. It was difficult because I had to rebrand online. And people of course know me because of my name online, and then I changed that to my normal name. That was a bit difficult, as it also felt like I was throwing something away. Some kind of success formula. But I had to do it. And that decision just gives me so much more peace now.” (Dagmar, follow-up interview, 08/11/22)*

Lisa expressed a desire to share her travel experiences but was constrained by the expectations of her existing followers. Consequently, she created a new Instagram account dedicated to her interests in travel and entrepreneurship. This allowed her to build up a new group of followers.

“*Well, I also started a second Insta [laughs]. Because I didn't have enough to do yet [laughs]. Yeah, because I really like entrepreneurship, traveling, things like that. But I can't share that on my other account, so I started a travel account. It already has 15,000 followers. And I also got my first collaboration with a sponsor.” (Lisa, follow-up interview, 08/12/22)*

**Figure 7:**
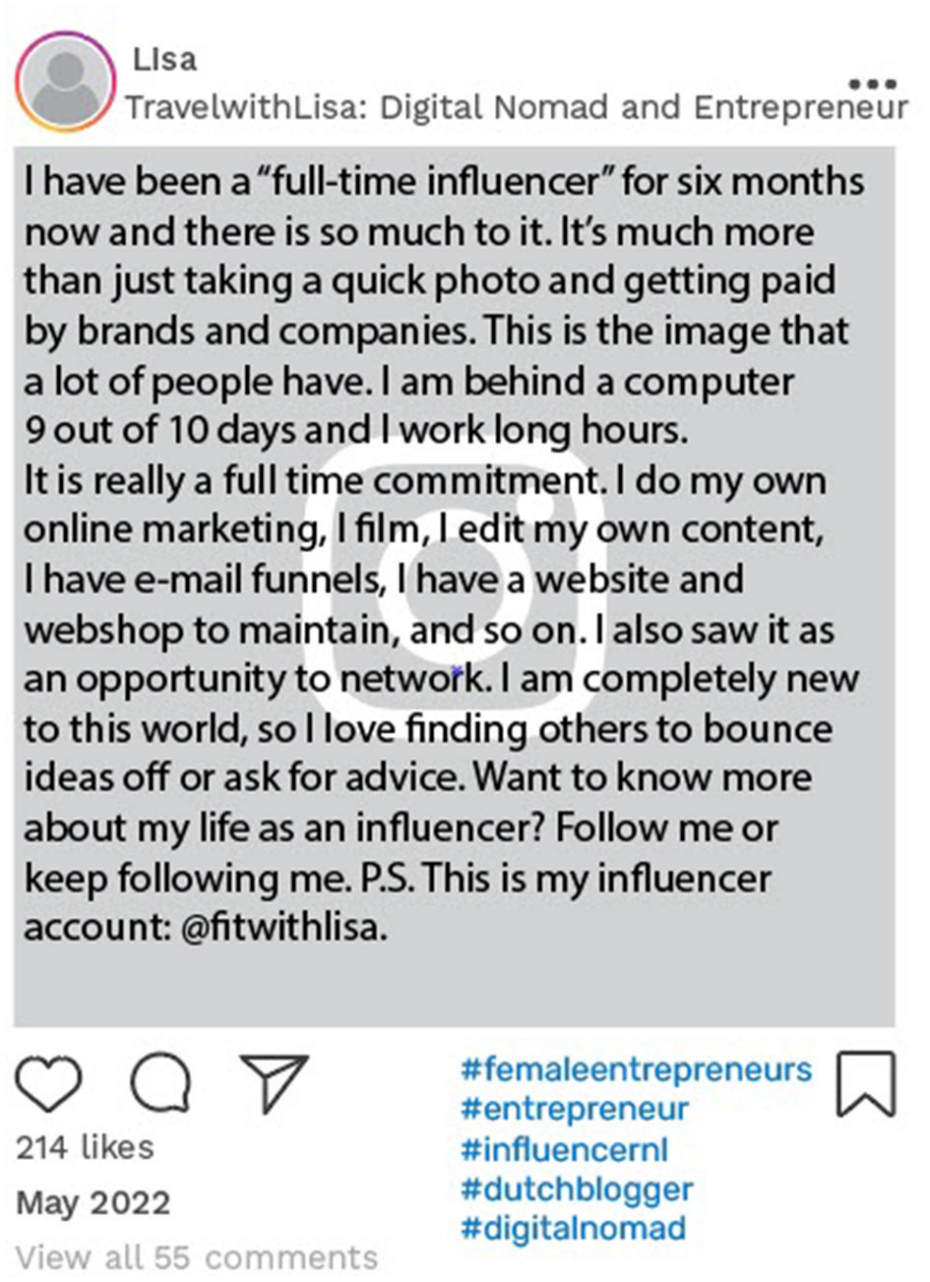
Example of online data taken from Lisa's Instagram account. Instagram posts designed and reproduced from s.salvador via Freepik (https://www.freepik.com/free-psd/instagram-post-template_5263426.htm#&position=1&from_view=author&uuid=2410d69f-1272-4628-a0b1-836d31b3bb20).

Lisa used one of her posts on her newly created account to explain the concept of being a social media influencer and to direct her followers to her primary influencer account. This post acted as an invitation to engage with her new content related to travel and entrepreneurship, ultimately fostering a social community around these topics, while directing her audience to her initial influencer account (Figure 7).

In general, some participants decided to add more identities through online identities because this seemed to be the best possible coping response to dealing with experiences of inauthenticity on their initial accounts. It seems apparent that for them, their online identities became firmly embedded in the expectations of their followers, making it difficult to fully disengage from these online identities. In response, they began to create and enact new online identities through different Instagram accounts, actively seeking validation from new audiences to support their evolving online identities while simultaneously lingering on their old accounts.

## 4 Discussion

In this study, we set out to expand the literature on identity work and online identities by identifying the challenges individuals perceive online and how they respond to those challenges over time by employing online identity work strategies and tactics. By taking a temporal perspective and rigorously studying seven extreme cases of Instagram micro-influencers using multi-method data, we were able to focus on online identity work dynamics over a longer period of time (Vantilborgh et al., 2018). Our results confirm the idea that online identity work can elicit challenges. We found that the interplay between amplified social expectations (Chae, 2018) and feelings of inauthenticity (Haimson and Hoffmann, 2016; Haimson et al., 2021), can lead to psychological distress (Primack et al., 2017; Berryman et al., 2018; Keles et al., 2020; Harren et al., 2021). Based on our findings, we argue that individuals' online identity work should be viewed as a dynamic process that changes over time. In particular, our empirical work suggests that in response to the challenges individuals may face online, they first enter a phase of experimentation, followed by either a phase of segmentation between online and offline identities, or a phase of adding identities through online multiplicity, i.e., using additional accounts to enact new online identities. Below, we will elaborate on the theoretical implications of our study.

First, our study further revealed how Instagram accounts can be considered as extreme liminal spaces (Stanko et al., 2022) that provide individuals with an environment between fantasy and reality where they can easily experiment with new behaviors and identities. Specifically, in response to the challenges they experienced, the individuals in this study first entered a phase of online experimentation. We suggest that during this phase, individuals can use their social media accounts as an “identity playground” (Stanko et al., 2019, 2022) that can be leveraged to explore desired identities and follower responses (Ibarra, 1999; Ibarra and Barbulescu, 2010). Much in line with the literature on identity play, defined as: “people's engagement in provisional but active trial of possible future selves” (Ibarra and Petriglieri, 2010, p. 11), we found how social media platforms can further help individuals to “freely experiment with the identity in question” (Ibarra and Barbulescu, 2010, p. 13) and gradually make changes to their online identity. We found that they may do this through online activation cuing and/or online relationship building. Identity research already suggested that identity markers, such as dress and other physical artifacts (Bataille and Vough, 2022), and building relationships through community participation (Stryker and Serpe, 1982) can cue the activation of new identities. Our research adds to the literature on identity work and online identities by showing how social media provides an environment where individuals can employ similar tactics to explore new identities on their online accounts. As a result, the online identity that is being experimented with can directly be evaluated by “likes,” “reposts,” and new “followers.” Particularly in the light of engagement analytics, individuals' identity work turns into a visible and measurable process online (Cover, 2015; Duffy et al., 2021).

Second, our results show that after experimenting, participants in this study chose between two different paths over time. They either further segmented their online and offline identities or added more identities through online multiplicity. Consistent with the authenticity paradox (Haimson et al., 2021), our findings showed that there were sometimes discrepancies between the participants' self-perceptions expressed during the interviews and their portrayals on their Instagram timelines (that is, the expression of a true self online) (Lim et al., 2015). Specifically, in some cases, the Instagram micro-influencers indicated that they had difficulty presenting a consistent self across online and offline contexts (Haimson et al., 2021), which led them to experience psychological distress. As a result, some may cope with the challenges they experience by explicitly creating clearer boundaries between their online and offline identities. Consistent with the literature on boundary work (Desrochers and Sargent, 2004; Ollier-Malaterre et al., 2013; Dumas and Sanchez-Burks, 2015), creating barriers between different identities (e.g., not displaying personal aspects on their online accounts or setting their account to private), may help individuals to reduce the identity conflict they experience. Here, we argue that the context of social media allows individuals to set clear limits on when and how many resources are dedicated to their online and offline identities (Bataille and Vough, 2022). That is, design elements in social media applications, such as privacy settings, help individuals to behaviorally segment different identities, which can help them reduce the identity conflict they experience. In sum, individuals may choose this pathway as it allows them to cope with the challenges they experience (e.g., identity conflicts and psychological distress) by putting clearer barriers between their identities.

However, instead of choosing the pathway of segmenting online and offline identities, some Instagram micro-influencers emphasized that they found it very difficult to cope with the challenges they faced. In particular, the social expectations that followers have online can exacerbate feelings of inauthenticity (Haimson et al., 2021), while at the same time meeting these expectations can further entrench an online identity in social interactions. Moreover, critical media studies have argued that for influencers in particular brand collaboration and monetization are key to their online success (e.g., Banet-Weiser, 2012; Duffy and Hund, 2015; Abidin, 2016). In this context, letting go of entrenched online identities can be a daunting task, despite strong feelings of inauthenticity. Our findings offer new insights by showing how individuals may find an escape from these feelings. Specifically, some individuals may decide to consciously create a new online identity on an alternate account, referred to as “adding identities through online multiplicity.” This pathway gives individuals a blank canvas on which they can redefine their online presence, without having to let go of their initial online identity. Our findings on online multiplicity bring forward insights on identity continuity (Wittman, 2019), specifically in online environments. Previous theories in the field of identity work have already explained that individuals often cling to former identities, even after changing roles (Wittman, 2019). Such a “lingering identity,” would for example be the musician who is unable to let go of his/her musician identity after experiencing a serious injury (Maitlis, 2009). Regardless of external audience perceptions, the musician would still identify as a musician because he/she would still feel like a musician. However, our findings suggest that individuals may cling to their online identities not necessarily because they still identify themselves as “fit girl,” but rather because they experience a strong sense of commitment to their online group of followers or sponsors and the expectations they may have of them (Stryker and Serpe, 1982, 1994). As a result, we argue that it is not a personal desire to hold onto previous online identities, such as the musician's experience, but rather the entrenchment of online social expectations that makes transitioning to a new online identity or letting go of a previous online identity a challenge. In sum, we add a more thorough consideration of identity continuity in online environments (Wittman, 2019). Specifically, we have seen that online identities may remain “sticky” even when individuals wish to move on. In other words, individuals may find themselves in a battle to make an identity change as they continue to identify themselves by an entrenched online identity, that is cultivated through sustained social interactions with engaged followers and corporate demands from the social media platform (Duffy and Hund, 2015). Here, the online identity work strategy of “adding identities through online multiplicity” may then provide them with an answer to the online authenticity paradox (Haimson et al., 2021), as the creation of a new online identity on a different Instagram account, may help individuals to make an identity change that presents a more consistent, positive, and “true” self without having to discard a certain status that was built within their original account.

This article makes a final methodological contribution to the literature on online identity work (Jäkälä and Berki, 2013; Cover, 2015; Haimson and Hoffmann, 2016; Barros et al., 2023) via our use of online autobiographical narratives revealed through the Instagram timelines of extreme cases (Eisenhardt, 1989). To date, various researchers in the field of identity work have emphasized the need to explore the temporal precedence of identity work to better understand its dynamics (Sveningsson and Alvesson, 2003; Knapp et al., 2013; Bataille and Vough, 2022). Our multi-method data, coming from interviews at two points in time and from Instagram timelines, help us to track online identity work over time. Online stories coming from Instagram timelines can be seen as powerful autobiographical accounts that help individuals to construct and share self-narratives in an online social context (McAdams, 1999; Stanko et al., 2022). Accordingly, we argue that these Instagram timelines are well suited to explore dynamic elements such as temporal patterns during alternating phases of identity stability and change.

### 4.1 Practical implications

We outline the following elements of online user experience design that can provide individuals with online spaces that can enhance their digital lifestyles.

#### 4.1.1 A space for the expression of authentic identities

To promote the expression of authentic selves in online environments (Haimson and Hoffmann, 2016; Haimson et al., 2021), the design of SNSs could consider certain elements of user experience design. One effective approach may be to configure applications that encourage users to present their true selves, potentially reducing identity conflict. Some SNS, such as BeReal and Everyday, have implemented this approach by encouraging users to share unfiltered moments from their daily lives. In addition, platforms such as Instagram and TikTok are beginning to adopt similar features in response to the growing trend of online authenticity. Nevertheless, it remains an open question whether these design features can fully challenge the prevailing culture of creating an “ideal online persona” and whether individuals will consistently find it difficult to present their authentic selves online (Duffy and Hund, 2015; Haimson et al., 2021).

#### 4.1.2 A space for community building

Online platforms could be further designed to enhance community building. As explained by earlier scholars, people's self-definitions and personal meanings are co-constructed through interaction with an audience (Goffman, 1959), and individuals are in need of communities where they have a sense of place and belonging (Baumeister and Leary, 1995). Social media can be used to find people with similar interests and join online communities with like-minded people. Some SNSs have already been designed around interest-based communities, such as UNBLND. UNBLND helps to connect strangers and connect them based on their interests and hobbies. As such, it becomes easier for individuals to find, for example, travel buddies, fitness buddies, or cooking buddies. Designed to build communities, apps can further help individuals to feel a sense of belonging, thereby allowing them to further express their authentic selves.

#### 4.1.3 A space for online multiplicity

Finally, online platforms could build in elements of user experience design that allow users to have multiple accounts on one platform, and easily switch between them (Haimson et al., 2021). In this way, users are better able to create boundaries between different online identities, which may help to reduce the conflict they experience. That is, by allowing multiple accounts, individuals can decide when and how many resources to devote to a certain online identity, which may also allow them to present a more coherent and consistent story on a given account. By segmenting different accounts and enabling online multiplicity, individuals can activate different parts of themselves with different interaction partners in different online environments. Although apps such as Instagram and Twitter allow individuals to have multiple accounts, many other apps make it difficult for users to switch between accounts (e.g., TikTok and SnapChat). These SNSs could further integrate features of online multiplicity, which could help individuals to switch between different accounts and to begin to build new online identities with different target audiences that they may perceive as more authentic. However, this design feature raises the question of whether having multiple accounts on a single platform may simultaneously increase the social expectations that are experienced, potentially leading to more psychological distress over time.

### 4.2 Limitations and future directions

The present study has some limitations, which suggest potential directions for future research. First, a limitation of our study is that our data was collected exclusively from seven extreme cases of Instagram micro-influencers on a single site (i.e., Instagram). While we believe that this group represents an extreme case for studying online identity work (Eisenhardt, 1989), and that Instagram provided us with a public platform that allowed us to explore visual content related to identity work, we also acknowledge that there is a potential bias in using this group as an extreme case. Aspiring or established influencers may be more aware of how Instagram's algorithm works and, as a result, may consciously tailor their online behavior to satisfy the algorithm, rather than express their authentic identities (Duffy et al., 2021; Haimson et al., 2021). The debate in critical media studies reflects the inherently paradoxical nature of the pursuit of authenticity in online spaces, and its ambivalent relationship with online success, brand collaboration, and monetization strategies (e.g., Boyd, 2010; Banet-Weiser, 2012; Marwick, 2013; Senft, 2013; Duffy and Hund, 2015; Abidin, 2016). Interestingly, our online observations revealed that those posts by Instagram micro-influencers in which they openly shared negative experiences, such as their struggles with mental health, resulted in the highest engagement metrics in terms of likes and comments, thus providing them with “positive quantified indices of visibility” (Duffy et al., 2021, p. 3). This observation raised an important question: does the act of sharing negative experiences truly stem from a desire to be authentic online, or does it serve as a strategic move to satisfy the algorithm and further capture the attention of fans and audiences? Furthermore, it could be argued that perceptions of online authenticity may also vary depending on the type of social media platform one is active on. That is, in this study we focused specifically on Instagram, a platform that is characterized by storytelling capabilities and a focus on sharing personal moments. What is considered authentic on Instagram, may not be the same on platforms like Twitter, blogs, or LinkedIn. Future research could consider expanding the sample to include a broader and more diverse range of social media users, such as young adults, or specifically those who use social media and experience psychological health problems. Additionally, future research could further explore how perceptions of authenticity might vary across platforms, and what identity work strategies are consequently employed on different platforms. Specifically, is there a difference between visual (e.g., Instagram) and text-based (e.g., Twitter) SNSs? To what extent do perceptions of authenticity differ between platforms where content may disappear quickly (e.g., SnapChat and Instagram stories) and those platforms where content may have a longer lifespan (e.g., YouTube or blog accounts)?

Second, to cope with the experienced challenges online we found that some individuals may add more identities through online multiplicity. They did so because they expressed struggling to let go of their online identities, which had been cultivated through sustained social interactions with engaged followers. Future research could explore the long-term effects of employing this strategy. That is, over the last decades, a multitude of studies in the field of multiple identities has examined the effects of multiple identities on health and wellbeing (Ramarajan, 2014). The conclusions of these studies, however, are conflicting. Some researchers take an “expansion” approach and suggest that having multiple identities (i.e., having higher self-complexity) would provide the individual with more resources and, hence, lead to better wellbeing (e.g., Carter, 2008). Others argue that having to navigate a multitude of identities may deplete time and energy due to more incompatible role behaviors or because of frequent transitions between these multiple identities (Kossek et al., 2012), which may lead to lower wellbeing (for a review see Barnett and Hyde, 2001). Even though online multiplicity was used here as a strategy to deal with experienced challenges, an interesting avenue for future research would be to better understand the long-term psychological consequences of online multiplicity.

Finally, we wish to conclude this section on limitations with a critical reflection on the results of our study and our perhaps “utopian” practical implications. Drawing on critical media studies (e.g., Boyd, 2010; Banet-Weiser, 2012; Marwick, 2013; Senft, 2013; Duffy and Hund, 2015; Abidin, 2016) and critical identity studies (e.g., Thornborrow and Brown, 2009; Brown, 2021; Barros et al., 2023), we would like to emphasize that SNSs are a product of a neoliberal capitalist society (Mukherjee and Banet-Weiser, 2012; Duffy et al., 2021). Therefore, SNSs are primarily designed with profit in mind. This profit motive is, in turn, the enemy of wide and sustained change. In particular, SNSs are powered by a neoliberal ethos of self-commodification (Duffy et al., 2021), and designed to encourage users to create and share content that can be monetized through advertising and data analytics (Mukherjee and Banet-Weiser, 2012). The algorithms are designed to keep the users engaged and to generate more interactions to provide more data for the platform (Bishop, 2018). Instagram micro-influencers in particular are highly susceptible to these normative and algorithmic controls (Barros et al., 2023), given that their online success is tied to metric success (Duffy et al., 2021). A number of studies in this vein have focused on how content creators, such as Instagram influencers, adapt their online brand (i.e., their online identity) to changes in the broader culture (e.g., Duffy et al., 2021), also referred to as consumerist identities (Banet-Weiser, 2012). Interestingly, in this study, for example, we saw that participants' online identities were closely aligned with societal trends in health and fitness, but slowly transformed to online identities that were more aligned with what the newspapers refer to as “spirituality and manifestation trends” (Haupt, 2021; Agg, 2022). These changes may be necessary for them to adapt their online brand to meet the needs of the online system and, in doing so, remain visible and successful (Duffy et al., 2021). Moreover, in line with critical media studies, we argue that algorithmic content creation can create echo chambers (i.e., online spaces in which individuals are primarily exposed to ideas, opinions, beliefs, or information that align with their existing beliefs and identities) that can further inhibit the authentic expression of the self. In these online echo chambers, individuals are exposed to content that consistently reinforces the beliefs and identities that they initially created online. The consistent reinforcement of beliefs and identities could potentially lead to what we call “sticky” identities in this paper; those identities that are difficult to let go of.

In closing, we call for additional critical insight into the complex interplay between the design of social media platforms within a neoliberal capitalist context, and the influence of this profit-driven design on online identity work. If we are committed to building a mental health friendly online world, we need to start working to change the profit motives of these online platforms. That is, as long as these platforms are designed with the idea of profit maximization in mind, the identity demands that these platforms impose on individuals will always perpetuate the challenges that individuals face online. In particular, echo chambers and algorithmic content curation may make it particularly difficult for individuals to explore and question their own beliefs and identities, possibly limiting the inherently dynamic process of identity work (Maitlis, 2009; Ramarajan, 2014; Caza et al., 2018; Bataille and Vough, 2022) and leading to mental health problems in the long run.

## 5 Conclusion

With the development of advanced media technologies, such as SNSs, individuals' identity work increasingly takes place online. While it could be argued that our sample of Instagram micro-influencers is “extreme” and “atypical” for studying online identity work, we contend that many people today experience similar challenges online and may cope with these challenges in comparable ways to the participants in this study. In particular, we expect that more recent groups of young individuals may have similar experiences, as it has been demonstrated that early adolescents are increasingly influenced by advanced media technologies, such as TikTok and SnapChat (Mittmann et al., 2022). Overall, we hope that this paper can spark interest in conducting further research on online identity work strategies in response to the challenges that individuals may face online.

## Data availability statement

The datasets presented in this article are not readily available because only excerpts/posts that have been used to underline the themes have been anonymized and included in the article. The rest of the dataset currently includes identifiable data. Requests to access the datasets should be directed to YB, bergs.y@buas.nl.

## Ethics statement

The studies involving humans were approved by the Research Ethics Committee Nyenrode Business Universiteit. The studies were conducted in accordance with the local legislation and institutional requirements. The participants provided their written informed consent to participate in this study.

## Author contributions

YB: Conceptualization, Formal analysis, Methodology, Writing – original draft, Writing – review & editing. PP: Conceptualization, Formal analysis, Supervision, Writing – review & editing. XL: Conceptualization, Formal analysis, Methodology, Supervision, Writing – review & editing. RB: Formal analysis, Methodology, Resources, Supervision, Writing – review & editing.
